# Density Functional Theory Calculations to Investigate the Role Played by an Aspartate Dyad in Hsp60-Catalyzed ATP Hydrolysis

**DOI:** 10.1021/acs.jpclett.5c02351

**Published:** 2025-10-10

**Authors:** Luca Torielli, Federica Guarra, Stefano A. Serapian, Giorgio Colombo

**Affiliations:** Department of Chemistry, 19001 University of Pavia, via Taramelli 12, 27100 Pavia, Italy

## Abstract

Adenosine 5′-triphosphate (ATP) hydrolysis is one of the most significant reactions in biochemistry. In chaperone proteins, energy released by hydrolysis enables them to carry out their function and help other proteins (called “clients”) to fold into their functional form. Here, we run Density Functional Theory calculations on three cluster models of the Hsp60 active site extracted from our previous molecular dynamics simulations of the 14-meric Hsp60 double-ring complex: our aim is to qualitatively investigate the mechanisms of ATP hydrolysis in different scenarios where the chaperone closes a dyad composed of catalytic aspartates Asp50 and Asp397. Since dyad closure raises Asp p*K*_a_ values and increases likelihood of protonation, we modeled the active site both in the presence and absence of a proton. Comparison of reaction barriers suggests that hydrolysis is favored when aspartates become deprotonated, explaining increased ATPase activity observed in V72I mutant Hsp60 (known to favor dyad closure).

## Graphical abstract

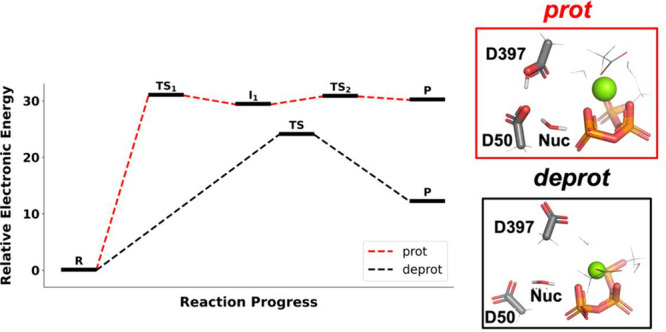

Hsp60 ^1,2^ is a chaperone protein that belongs to the family of Group I chaperonins. These chaperones play a crucial role in maintaining proteostasis, both under physiological conditions and in stressed environments.^3^ Dysregulation of Hsp60 function has been linked to various pathological conditions, particularly Alzheimer’s disease and cancers.^4,5^ Consequently, interest in Hsp60 has grown over the years, as has the drive to understand its functionality and structure.

Group I chaperonins^6,7^ rely on the hydrolysis of adenosine 5′-triphosphate (ATP) hydrolysis to adenosine 5′-diphosphate (ADP) and inorganic phosphate (P_i_) to carry out their function. The reaction is in fact coupled to the structural remodeling that leads to the folding of “client” proteins inside a confined folding chamber arising at the core of multimeric self-assemblies.

Under physiological conditions, Hsp60 cycles through several forms.^8–10^ It can appear as a heptameric single-ring complex **S** containing a central cavity to host the client(s). A double-ring complex **D** consists of two units of **S** interfaced at their equatorial domains (*vide infra*). Finally, **D** can recruit 7 units of cochaperone protein Hsp10 at each pole (14 in total), resulting in football-shaped complex **F** (Figure 1a), whose central chamber is sealed off and ready for client folding upon hydrolysis of the 14 ATP molecules bound to **F** (one per Hsp60 monomer).

**Figure 1:**
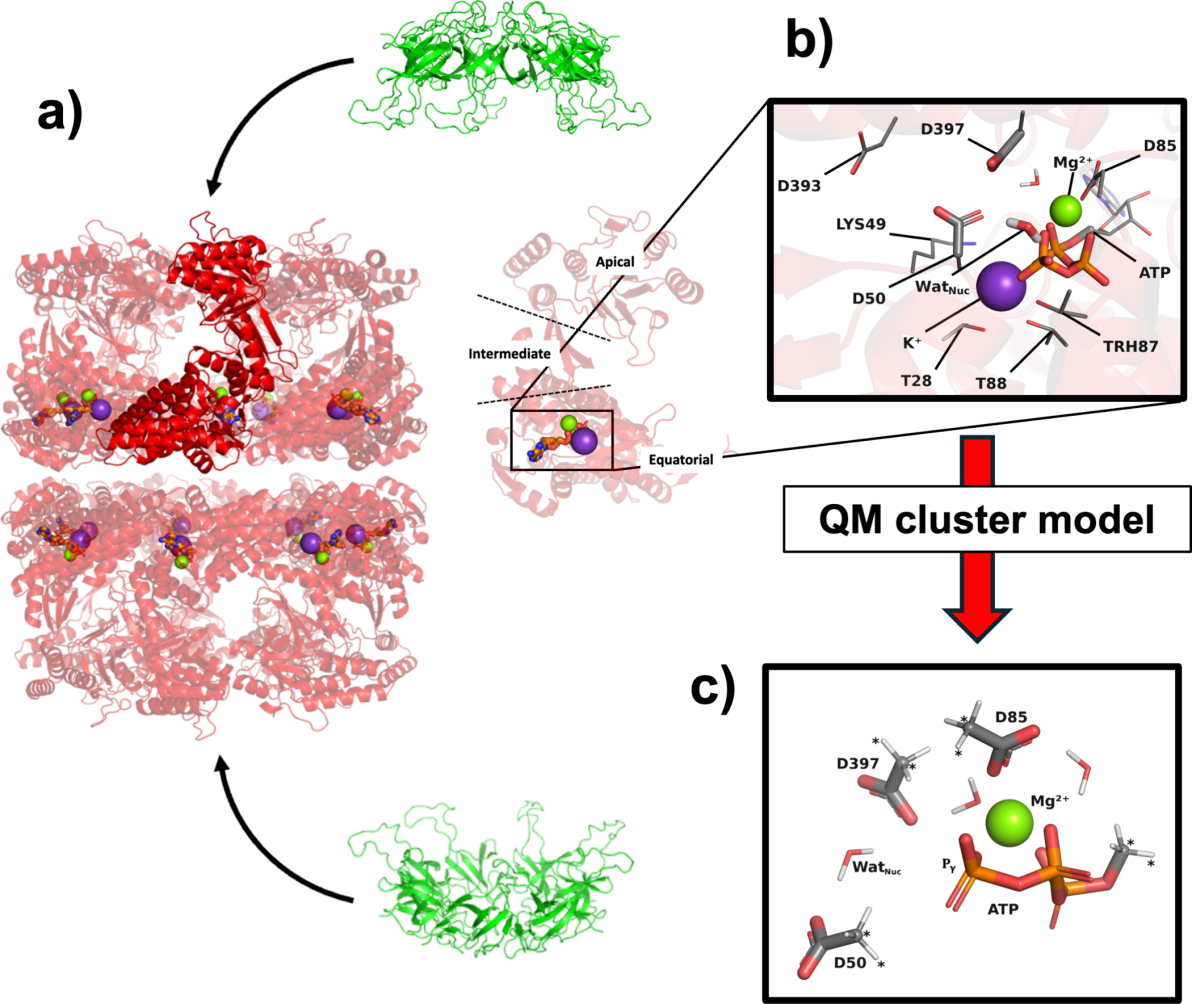
a) Formation of football-shaped complex **F** by 14 monomers of Hsp60 (red) arranged as a double ring **D** and 14 monomers of cochaperone Hsp10 (green). A single Hsp60 monomer **M** is shown with full opacity for reference. A separate instance of **M** is shown immediately on the right (whose apical, intermediate, and equatorial domains are labeled). b) Zoom on the active site of one Hsp60 **M**, with key catalytic actors labeled. Heavy atoms rendered as sticks and Mg^2+^ (green sphere) are atoms included in our cluster models. Atoms rendered as lines and K^+^ (purple sphere) atoms are other catalytically relevant actors not included in the models. Hydrogens are omitted for clarity, except on two water molecules, one of which is nucleophilic water Wat_Nuc_. c) Active site atoms considered in our cluster models (taken from another snapshot), including capping H atoms to saturate dangling valencies. Atoms marked with an asterisk were kept frozen throughout the optimization process. Color code for all panels: Mg^2+^ (green spheres) and K^+^ (purple spheres); C, O, N, P, H in gray, red, blue, orange, and white, respectively.

Each Hsp60 monomer **M** in **S**, **D**, and **F**, comprises three structural domains: equatorial, apical, and intermediate (Figure 1b). Most of the active site (Figure 1b) is contained in the equatorial domain of each **M**, including the highly conserved^11,12^ Asp50: this is one of two catalytic aspartates prone to deprotonate the nucleophilic water molecule Wat_Nuc_ that attacks the Pγ atom of ATP (labeled in Figure 1c). A C-terminal region of the equatorial domain is also involved in client binding—pointing toward the interior of the folding chamber in **D** and **F**—and the domain also fundamentally mediates inter- and intraring interactions.

One key catalytic protagonist that remains outside the equatorial domain is a second aspartate (Asp397), also conserved,^11,12^ which is found on helix α14 in the intermediate domain and forms a dyad with Asp50 (*vide infra*). Finally, the apical domain (Figure 1b) mediates interactions with cochaperone Hsp10 in **F**, and, again, the enchambered client.

We have recently^13^ reported a series of comparative molecular dynamics (MD) simulations of **M**, **S**, **D**, and **F** of wild-type (WT) Hsp60 and mutant V72I. The latter variant is associated with a form of hereditary spastic paraplegia, and with respect to WT Hsp60, it shows oligomer stabilization, impaired client folding ability, and a subtle increase in ATPase activity.^13–15^ We found that the V72I mutation, which is not adjacent to ATP despite being in the equatorial domain, significantly rewires allosteric communication pathways present in WT Hsp60. In the WT, the intermediate domain acts as a hinge region that controls the raising of the apical domain to capture Hsp10, and the lowering of Asp397 on helix α14 to “close” the catalytic dyad. Mutation V72I manages to disrupt allosteric communication across the intermediate domain, resulting in decoupling of the apical domain from the equatorial domain. Intriguingly, we also observed that in mutant assemblies containing ATP (**D** and **F**), Asp50 and Asp397 are able, albeit in a fraction of cases, to come closer by about 0.25 Å (as measured at their Cγ atoms) than they are ever able to do in WT Hsp60.

Guided by chemical intuition, we speculated that the slightly better ability of the mutant to push Asp50 and Asp397 closer together despite their negative charge might be behind the increase in reactivity: the p*K*_a_ of 8.22/12.52 (Asp50/Asp397) predicted by the PropKa package^16^ was certainly higher than the 3.18/4.91 predicted in **M** and **S** (where ATP is absent and the dyad is more open), implying enhanced basicity. However, we did not have the opportunity of validating this hypothesis, for example by proving that increased dyad basicity leads to easier deprotonation of Wat_Nuc_ or, that if a proton were captured by the more basic dyad from elsewhere as seems to be the case in starting CryoEM structures of **D** and **F** (PDB: 8G7L and 8G7N),^10^ the reaction could still proceed more favorably even with the catalytic dyad not fully preorganized.

Here, to qualitatively shed light on possible ATP hydrolysis mechanisms in Hsp60 and on the role of protonation in the catalytic Asp50-Asp397 dyad, we trace the potential energy surface (PES) for ATP hydrolysis by conducting Density Functional Theory (DFT) calculations on three simplified cluster models^17,18^ of the Hsp60 active site, excided from our classical MD simulations of WT and V72I **D** (see, e.g., Figure 1c). To ensure as fair a comparison as possible, the PES for hydrolysis is traced in the same cluster models, optimized in both the presence and absence of a shared proton between the catalytic aspartates. This choice ensures that the degrees of freedom that change the most in the absence of a proton are those strictly associated with deprotonation (i.e., side chains and H_2_O that are involved in hydrogen bonds in the protonated system), while optimization of the remaining degrees of freedom results in minimal changes from the protonated to deprotonated systems.

## Generation of Six Cluster Models from Three Classical MD Poses

Table 1 provides details on the three classical MD simulation poses of **D** ^13^ (Figure S1) from which we excided three initial cluster models^17,18^ of the active site of one of the Hsp60 monomers containing Mg^2+^-chelated ATP. Each model was optimized at the B3LYP/6-31G­(d) level^19,20^ of Density Functional Theory (DFT) (see Methods for more details). We henceforth label these models **A**_**prot**_ to **C**_**prot**_, since all three originate from Hsp60 monomers coming from MD simulations of **D** with protonated Asp397. Models are illustrated in the left column of Figure 2 in their initial reactant state, **Reac**, in which ATP is ready to be attacked by a nucleophilic water molecule Wat_Nuc_ that is in turn poised for deprotonation by either Asp50 or Asp397.

**Table 1:** Classical MD Simulation Poses of **D** from Which the Three Initial Protonated Cluster Models Featured in This Study Were Excided

***Cluster Model***	***Replica (/4)***	***Frame (/10000)***	***Protomer (/14)***	***Variant***
A_prot_	1	1482	13	V72I
B_prot_	4	2428	2	V72I
C_prot_	1	386	7	WT

**Figure 2:**
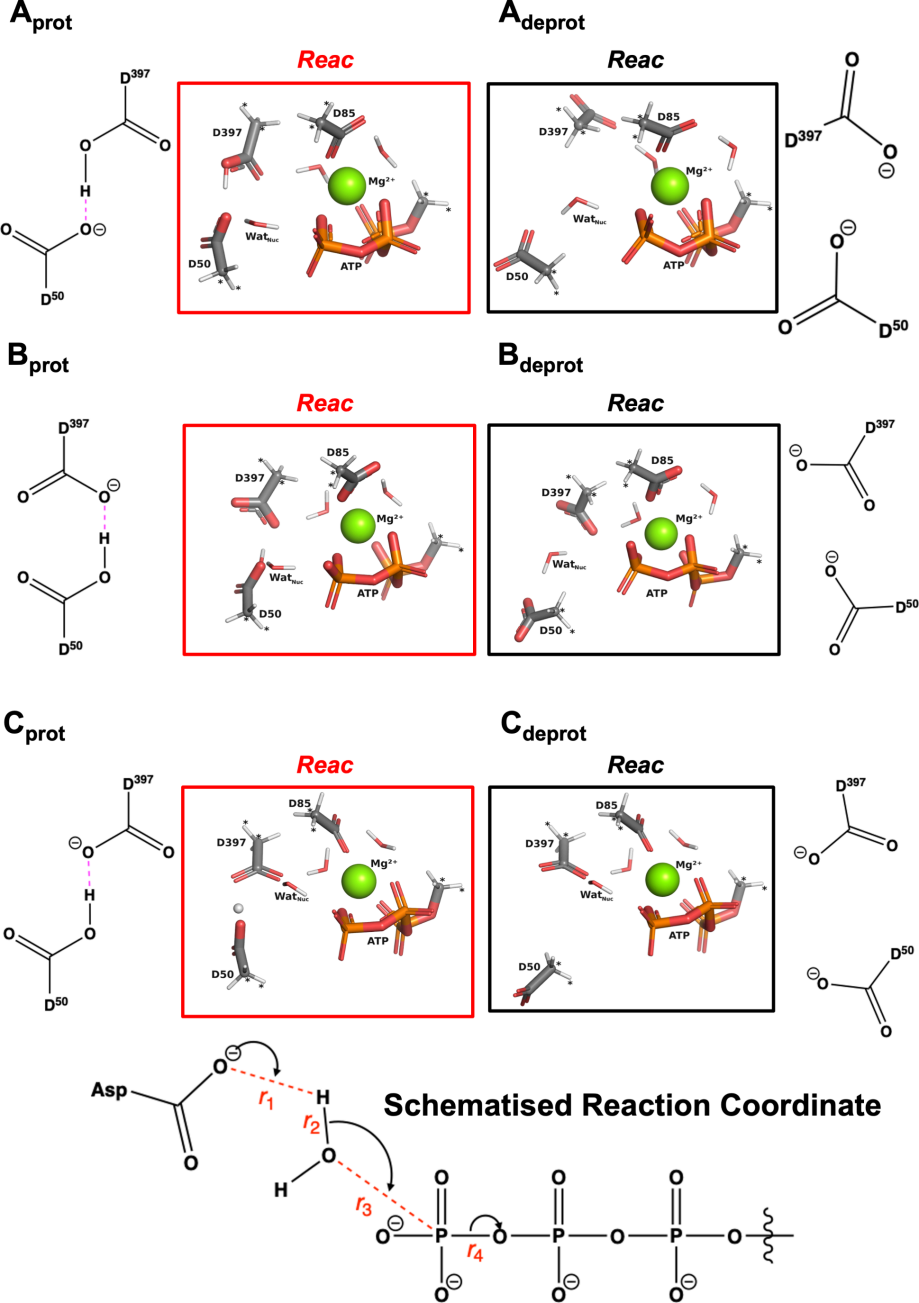
Top: (Red frames; left) protonated cluster models of the active site **A**_**prot**_, **B**_**prot**_, and **C**_**prot**_, in their **Reac** state. (Black frames; right) deprotonated cluster models of the active site **A**_**deprot**_, **B**_**deprot**_, and **C**_**deprot**_, in their **Reac** state. All six cluster models are optimized at the B3LYP/6-31G­(d) level of DFT; orientation of the catalytic dyad in each model is also shown in 2D next to each frame, with hydrogen bonds for protonated clusters marked in magenta. Atoms marked with “*” were kept frozen throughout the optimization process. Color code: Mg^2+^ as green spheres; C, O, P, H in gray, red, blue, and white, respectively. Bottom: schematized reaction coordinate (RC) used to determine MD poses in a “reactive” state (see main text for details), i.e., poses with atoms in a favorable geometry for ATP hydrolysis to begin. Reaction progress is a sum of phosphate transfer (*r*_4_ – *r*_3_) and proton transfer (*r*_2_ – *r*_1_); distances are marked in red. Present in the bottom scheme are the carboxylate group of either Asp50 or 397 (D50 or D397), Wat_Nuc_, and ATP phosphates α, β, and γ.

While the V72I mutation is too far away to be included explicitly in our cluster models (further discussed in Methods and as Supporting Information), its allosteric effects—including facilitation of catalytic dyad closure compared to WT—have been adequately^13^ included during our preceding MD simulations. For this reason, MD poses listed in Table 1 come indistinctly from MD simulations of WT and V72I. Cluster models generated from these poses should be considered as having the effects of the V72I mutation implicitly incorporated: while poses similar to the first two entries in Table 1 also arise in MD simulations of WT **D** (see, e.g., Table S1 and Figure S2), and poses similar to the last entry in Table 1 are also found in MD simulations of V72I **D** (example in Table S1 and Figure S2), what counts toward reactivity are the different proportions in which related poses are collectively sampled during the simulation.

**A**_**prot**_, **B**_**prot**_, and **C**_**prot**_ were deliberately chosen from MD poses (Figure S1) in which one of the Hsp60 monomers featured the catalytic Asp50/Asp397 dyad in different arrangements (deemed to be chemically representative of water activation for ATP hydrolysis). In addition, it was required that the active site of the monomers in question retained a “reactive” configuration (explained in more detail further below).

Original dyad arrangements were as follows. The parent pose of **A**_**prot**_ (Figure S1, top) featured protonated Asp397 hydrogen-bonded to the same Asp50 oxygen that was prone to deprotonate Wat_Nuc_. The pose generating **B**_**prot**_ (Figure S1, middle) is similar, but in addition, the protonated oxygen of Asp397 is also hydrogen-bonded to one of the H_2_O molecules coordinating the chelated Mg^2+^; the counterpart isolated from MD simulations of WT **D** (Figure S2; top) shares the same characteristics. Finally, cluster model **C**_**prot**_ (Figure 1, bottom) was isolated from a pose in which Wat_Nuc_ is prone to be deprotonated not by Asp50 but by Asp397 and is oriented toward the latter’s nonprotonated oxygen; we chose this pose to test a case in which Asp50 sequesters the proton from Asp397, and the two electrons in turn drive Wat_Nuc_ deprotonation. Again, we observe similar poses in MD simulations of V72I **D** (Figure S2; bottom).

In reality, the switch from a classical force field to DFT and the concomitant removal of some active site features already lead to a few significant changes compared to classical MD (cf. Figure S1 and the left-hand side of Figure 2). In **B**_**prot**_, for example, DFT optimization spontaneously shifts the proton from Asp397 to the Asp50 oxygen that should deprotonate Wat_Nuc_, creating an even more interesting situation to test. In **C**_**prot**_, we observe a similar proton transfer upon DFT optimization, this time possibly making Asp397 more reactive. Still, models **A**_**prot**_, **B**_**prot**_, and **C**_**prot**_ in their **Reac** state retain Asp50/Asp397 in sufficiently different and chemically representative orientations.

As stated, when searching for specific dyad arrangements across MD poses of **D**, we also had to make sure that Hsp60 active sites eventually chosen for cluster model construction were “reactive”. First of all, a “reactive” active site (see e.g., refs 21 and 22) is one that retains all other electronically important actors in an appropriate structural arrangement (Supporting Information) since, without these conditions, ATP hydrolysis would not be catalyzed in the first place. Actors include a K^+^ present in most ATP crystal structures that is required for catalysis,^23^ and several conserved residues.^11,12,24^ Second, we chose active sites in which the general reaction coordinate describing ATP hydrolysis (RC; schematized in Figure 2 at the bottom) was as close as possible to 0 (i.e., poses with geometries as favorable as possible for the hydrolysis reaction to kick off). In line with a previous ATP hydrolysis study,^25^ the RC is expressible as (*r*_2_ – *r*_1_) + (*r*_3_ – *r*_4_), where *r*_2_ – *r*_1_ measures the extent of proton transfer; *r*_3_ – *r*_4_ expresses the extent of phosphate transfer; and *r*_1_ to *r*_4_ are the individual bonds or distances (labeled in Figure 2, and typically reported in Å). Therefore, prior to the reaction, *r*_2_ < *r*_1_ and *r*_3_ < *r*_4_, meaning that RC is always negative to begin with and ends up positive.

In turn, **A**_**prot**_, **B**_**prot**_, and **C**_**prot**_ also served as starting points to generate three additional reoptimized cluster models **A**_**deprot**_, **B**_**deprot**_, and **C**_**deprot**_, this time with both aspartates deprotonated: we show their DFT-optimized **Reac** state in the right column of Figure 2. Removal of the proton in **A**_**deprot**_ leads to dyad disruption upon DFT optimization, with the newly created −1 charge pushing Asp397 away. Reoptimization of **B**_**deprot**_ shows an ideal situation in which dyad aspartates manage to stay close, and each Wat_Nuc_:H points to an Oδ of a different Asp. When reoptimizing **C**_**deprot**_, Wat_Nuc_ remains hydrogen-bonded to Asp397 like in the protonated counterpart, and this time it is Asp50 that is pushed away.

More details on the generation of **A**_**deprot**_, **B**_**deprot**_, and **C**_**deprot**_ from **A**_**prot**_, **B**_**prot**_, and **C**_**prot**_, respectively, are provided in the Methods section, along with details of confirmatory reoptimizations run at the B3LYP/6-311++G­(2d,2p) level on larger cluster models featuring K^+^ and three active site threonines (structures in Figure S3; energies in Figure S4).

## PES Profiles for ATP Hydrolysis

Proceeding from cluster models **A**_**prot**_, **B**_**prot**_, **C**_**prot**_, **A**_**deprot**_, **B**_**deprot**_, and **C**_**deprot**_ in their **Reac** state, we mapped the potential energy surface (PES) characterizing ATP hydrolysis in all six scenarios. Potential energy profiles at the B3LYP/6-31G­(d) level are mapped in Figure 3: PESs for deprotonated and protonated versions of the same cluster model are mapped within the same panel. All hydrolysis paths go from reactant state **Reac** to product **Prod** passing through sequentially numbered first order transition states **TS**_**x**_ and, if present, intermediates **Int**_**x**_. As a note of advice, we should mention that our definition of a **Prod** state entails full transfer of a proton from the attacking Wat_Nuc_ to either Asp50 or Asp397 and formation of ADP + [HPO_4_]^2–^ or, in the case of **C**_**prot**_, its Asp397-mediated transfer to another inorganic phosphate oxygen, resulting in ADP + [H_2_PO_4_]^−^.

**Figure 3:**
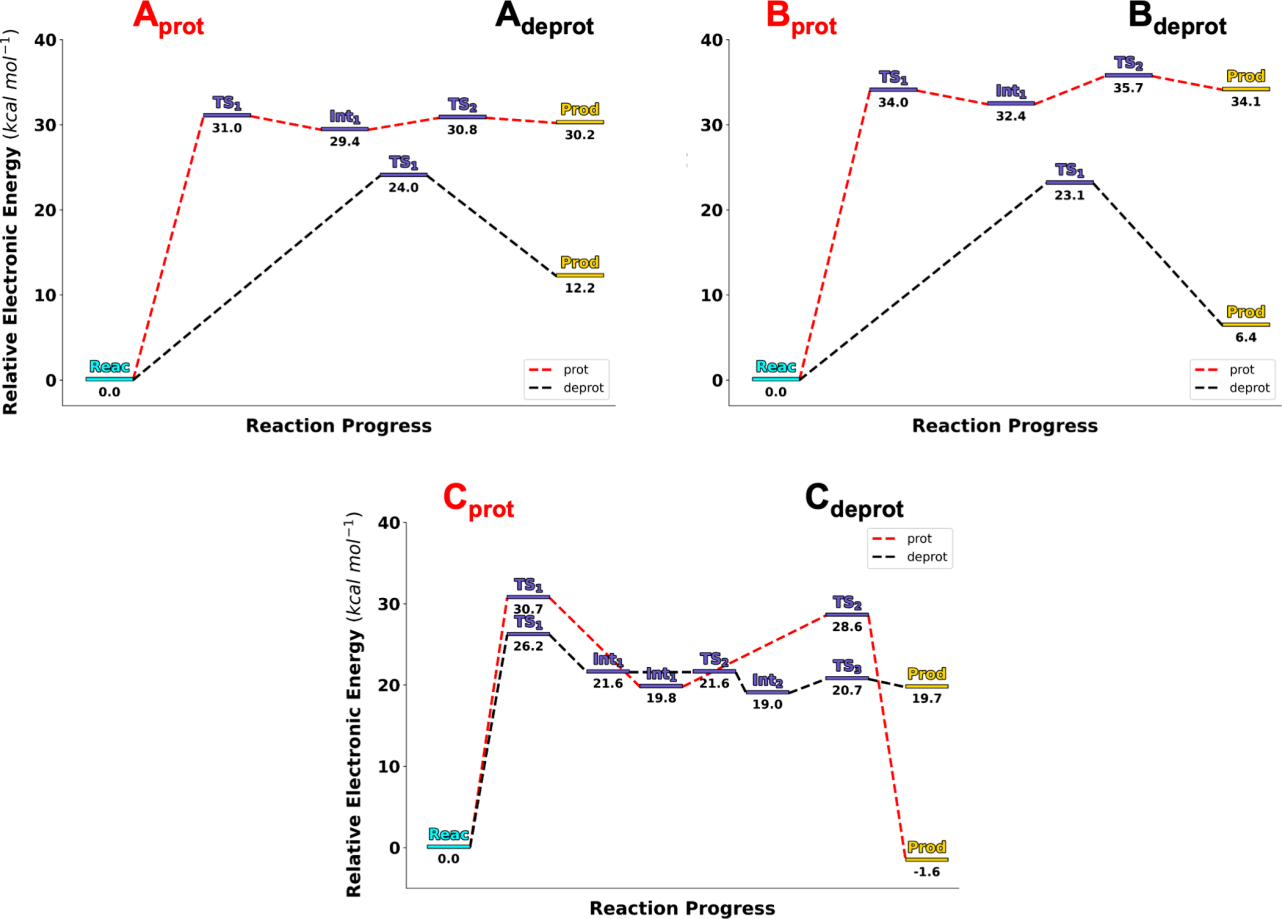
Electronic energy profiles for the complete progress from **Reac** to **Prod** in all six cluster models featured in our study, at identical scales, as measured at the B3LYP/6-31G­(d) level of theory relative to that of **Reac**. Protonated (red profiles) and deprotonated (black profiles) versions of cluster models generated from poses **A**, **B**, and **C** (Table 1) have been grouped into three different panels, respectively (two cluster models *per* panel). Stationary points marked in the profiles are illustrated in Figures 4 and 6.

In the following paragraphs, we provide a more detailed account of each path to full ATP hydrolysis, commenting on individual PESs and stationary points. We will discuss structures and energies at the B3LYP/6-31G­(d) level, focusing on the (otherwise similar) confirmatory calculations at the B3LYP/6-311+G­(2d,2p) level mainly when there are discrepancies. All calculations and resulting geometries are available online, on the *ioChem-BD* server^26^ (Link: 10.19061/iochem-bd-6-535).

## ATP Hydrolysis in A_prot_ and A_deprot_

Figure 4 shows all salient stationary points along the path to ATP hydrolysis in cluster models **A**_**prot**_ and **A**_**deprot**_ (i.e., it is associated with two PES plots shown, respectively, in red and black in Figure 3a). In **A**_**deprot**_ removal of the proton leads to a **Reac** state (Figure 2; top right), in which the newly created extra negative charge has pushed Asp397 away.

**Figure 4:**
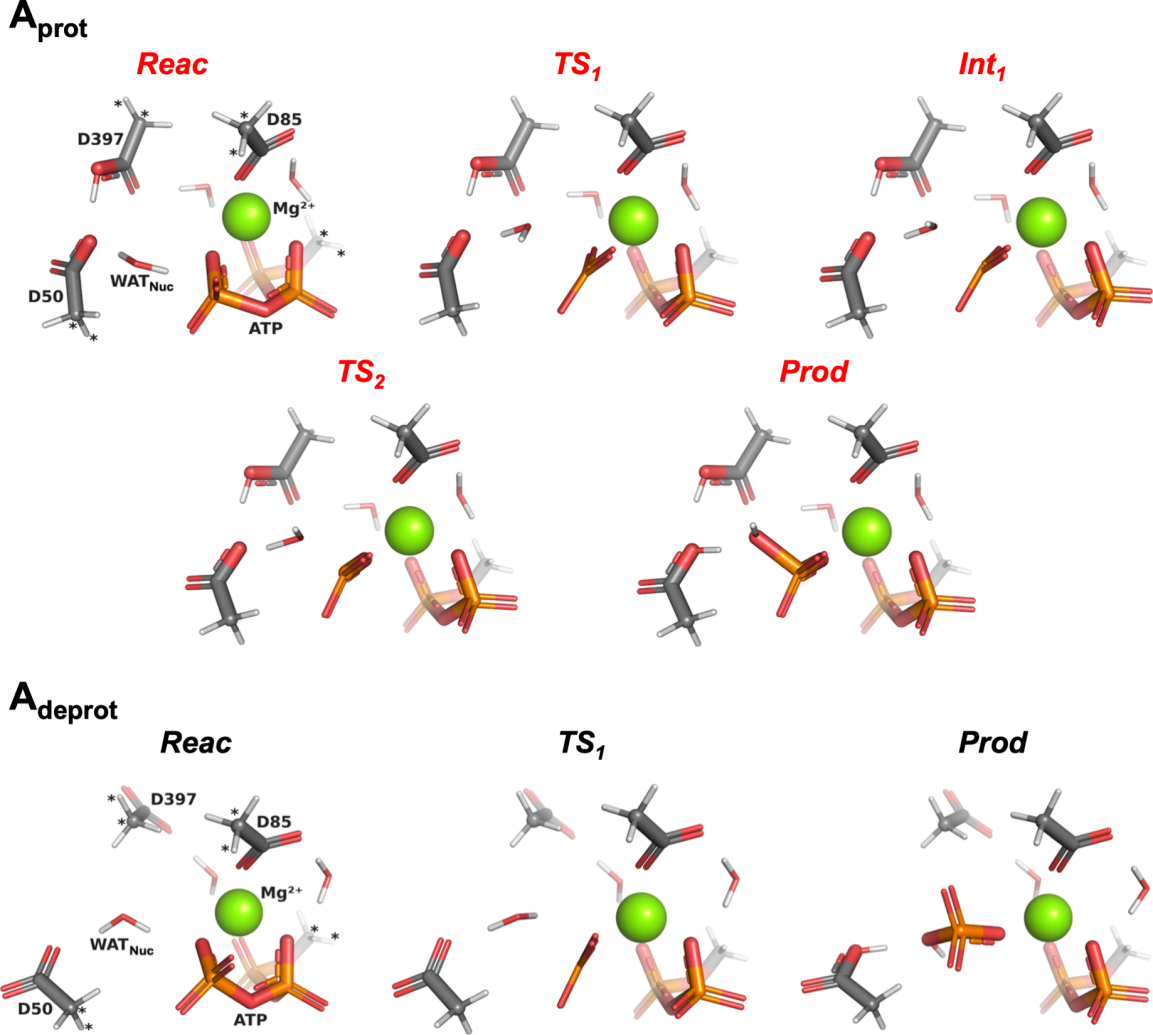
Stationary points (intermediates **Int**_**x**_ and first-order transition states **TS**_**x**_) encountered along the progress from **Reac** (cf. Figure 2) to **Prod** in cluster model **A**_**prot**_ (red; top) and cluster model **A**_**deprot**_ (black; bottom), optimized at the B3LYP/6-31G­(d) level. The color code is the same as in Figure 2. Bonds have been automatically drawn by the *PyMOL* suite^27^ based on interatomic distances. Atoms marked with “*” were kept frozen throughout the optimization process. All optimizations and final geometries are available online (see main text).

The first aspect that emerges when comparing PESs in Figure 3a is that ATP hydrolysis is still clearly favored in **A**_**deprot**_ compared to **A**_**prot**_; this is despite the dyad becoming disbanded and Asp50 left to act on its own as base. Indeed, in **A**_**deprot**_, the reaction occurs in a single step whose barrier is notably lower than that for the rate-determining step in **A**_**prot**_ (**TS**_**1**_; ΔΔ*E* = −7.0 kcal mol^–1^). The single **TS**_**1**_ characterizing hydrolysis in **A**_**deprot**_ (labeled in Figure 4) clearly resembles those observed in other mechanistic studies (e.g., refs 25), with a planar metaphosphate species (Pγ–Oβ bond cleavage prior to Wat_Nuc_:O–Pγ bond formation), and proton transfer occurring asynchronously just after the barrier. Conversely, in **A**_**prot**_, besides the aforementioned higher energy barrier for the rate-determining step (**TS**_**1**_; Pγ–Oβ bond cleavage), the reaction takes place in two separate steps: proton transfer to Asp50 is not barrierless—i.e., not concerted with phosphate cleavage. Rather, there arises an intermediate **Int**_**1**_ trapped at +29.4 kcal mol^–1^ in which Wat_Nuc_ still has not been able to pass one of its protons to Asp50. This comes subsequently (**TS**_**2**_), at a cost of +1.4 kcal mol^–1^. In the enlarged version of **A**_**prot**_ (Figures S3 and S4), the barrier for proton transfer to Asp50 disappears, directly affording **Prod**; nonetheless, the cost for phosphate cleavage (**TS**_**1**_) compared to **A**_**deprot**_ rises considerably.

## ATP Hydrolysis in B_prot_ and B_deprot_

Salient stationary points encountered on the path to ATP hydrolysis in **B**_**prot**_ and **B**_**deprot**_ are shown in Figure 5, whereas the potential energy profiles are indicated in Figure 3b. We recall that in **Reac** in **B**_**prot**_, the proton is transferred to Asp50 instead of Asp397 like in **A**_**prot**_, and Asp397 itself—now the apparent base despite being unbonded to Wat_Nuc_—is engaged in an additional hydrogen bond with a H_2_O molecule coordinating Mg^2+^. **Reac** in **B**_**deprot**_ is even more notable because, thanks to the distinctive orientation of Wat_Nuc_ that sandwiches between the Asp50/Asp397 and orients its oxygen toward Pγ, it is the only deprotonated model in which both catalytic aspartates remain oriented toward each other. This asset is conserved in the enlarged version of **B**_**deprot**_ (Figure S3), even though the **TS**_**1**_ we obtain has Wat_Nuc_ temporarily losing its hydrogen bond to Asp397.

**Figure 5:**
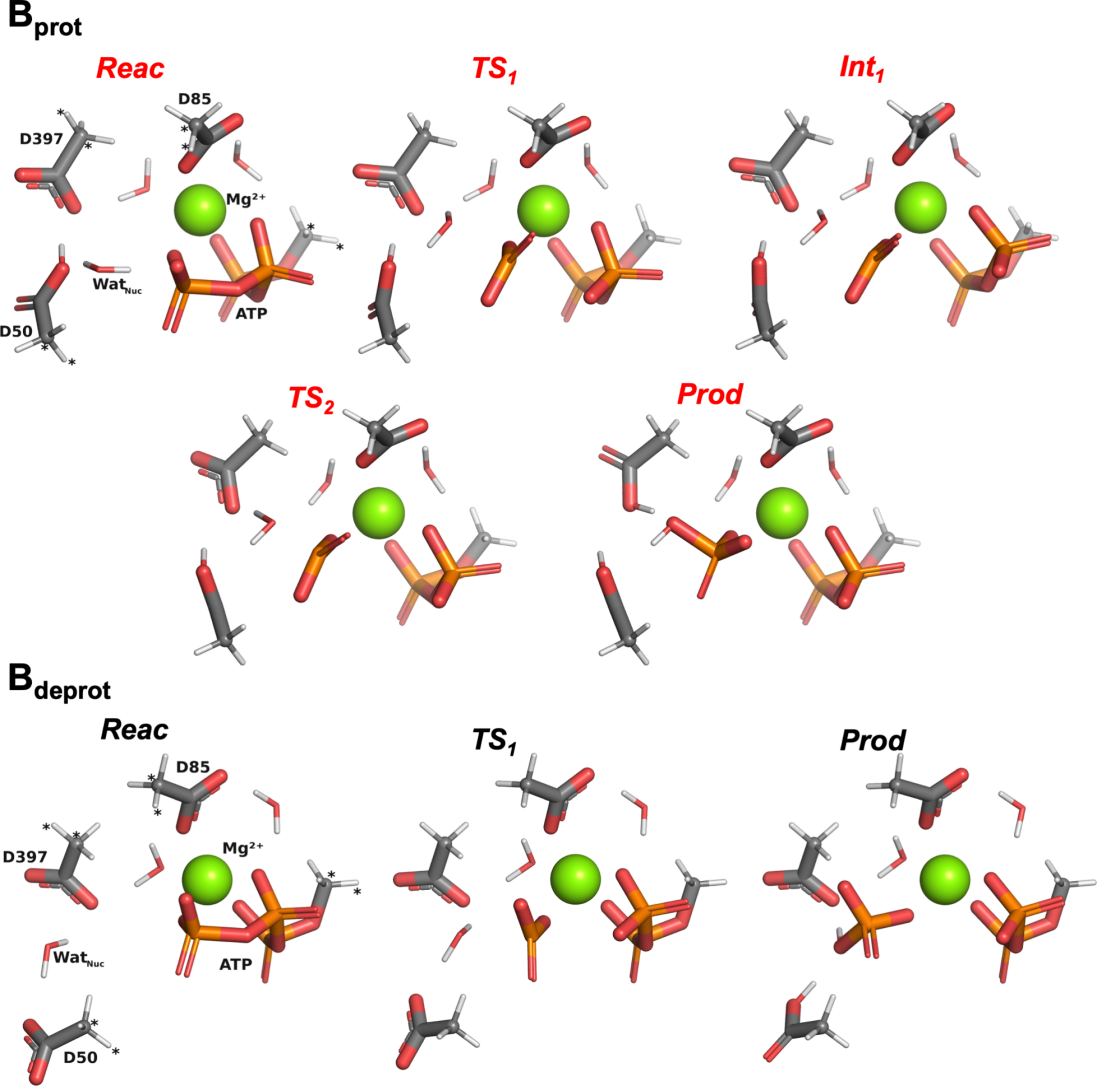
Stationary points (intermediates **Int**_**x**_ and first-order transition states **TS**_**x**_) encountered along the progress from **Reac** (cf. Figure 2) to **Prod** in cluster model **B**_**prot**_ (red; top) and cluster model **B**_**deprot**_ (black; bottom), optimized at the B3LYP/6-31G­(d) level. The color code is the same as in Figure 2. Bonds have been automatically drawn by the *PyMOL* suite^27^ based on interatomic distances. Atoms marked with “*” were kept frozen throughout the optimization process. All optimizations and final geometries are available online (see main text).

Whereas ATP hydrolysis in **B**_**deprot**_ features an even lower barrier compared to **A**_**deprot**_ (+23.1 kcal mol^–1^ instead of +24.0 kcal mol^–1^), this decrease does not hold in the larger versions of **A**_**deprot**_ and **B**_**deprot**_ (Figure S4), whereby hydrolysis in the former remains significantly cheaper than in the latter (22.6 kcal mol^–1^ vs 29.8 kcal mol^–1^). Structurally, trends with respect to **A**_**deprot**_ are conserved: in both the smaller and larger versions of **B**_**deprot**_, hydrolysis occurs in a single step, whose **TS**_**1**_ (Figure 5) is structurally very similar to **TS**_**1**_ in **A**_**deprot**_.

At ΔΔ*E* = −12.6 kcal mol^–1^, the difference in cost compared to hydrolysis in **B**_**prot**_ is even more substantial than the one observed between **A**_**deprot**_ and **A**_**prot**_ (with the trend this time confirmed in the larger versions; Figure S4). Like in **A**_**prot**_, ATP hydrolysis in **B**_**prot**_ is also characterized by two barriers **TS**_**1**_ (phosphate cleavage) and **TS**_**2**_ (proton transfer) that are separated by a high-energy intermediate **Int**_**1**_. However, both **TS**_**1**_ and **TS**_**2**_ bear even higher costs compared to **A**_**prot**_ (ΔΔ*E* = +3.0 and +4.9 kcal mol^–1^, respectively). This is in line with what chemical intuition would suggest: Asp397 has a higher number of hydrogen bonds and, as shown by the initial DFT optimization of **Reac**, it is not strong enough to deprotonate Asp50 so that it can act as the base. **TS**_**1**_ carries the cost of rearranging Wat_Nuc_ so that it hydrogen-bonds to Asp397; **TS**_**2**_ carries the cost of the poor basicity of Asp397.

## ATP Hydrolysis in C_prot_ and C_deprot_

In **C**_**deprot**_, optimization of **Reac** again leads to dyad disbandment, but this time, it is Asp50 that moves away and Asp397 that is poised to become the base: Asp397 remains hydrogen-bonded to Wat_Nuc_ and, additionally to one of the Mg^2+^ coordination waters. Hydrolysis costs compared to **C**_**prot**_ are—unlike for models **A**_**prot**_/**A**_**deprot**_ and **B**_**prot**_/**B**_**deprot**_—significantly more modest (Figure 3c; Figure S4): ΔΔ*E* is a mere −4.6 kcal mol^–1^ in the smaller, B3LYP/6-31G­(d)-optimized cluster model and as small as −0.5 kcal mol^–1^ in the larger B3LYP/6-311++G­(2d,2p)-optimized version. Furthermore, in the smaller version of **C**_**deprot**_, proton transfer is seen to take place over several steps (Figure 6; energies in Figure 3c), in a way that is reminiscent of protonated models: after the phosphate cleavage step **TS**_**1**_. It takes the enlargement of **C**_**deprot**_ to include K^+^, Thr28, Thr87, and Thr88 and expansion to the 6-311++G­(2d,2p) basis set to restore a barrierless H^+^ transfer to Asp397 (Figures S3 and S4), likely aided by the emergence of a hydrogen bond between Thr87 and one of the P_i_:Oγ atoms.

**Figure 6:**
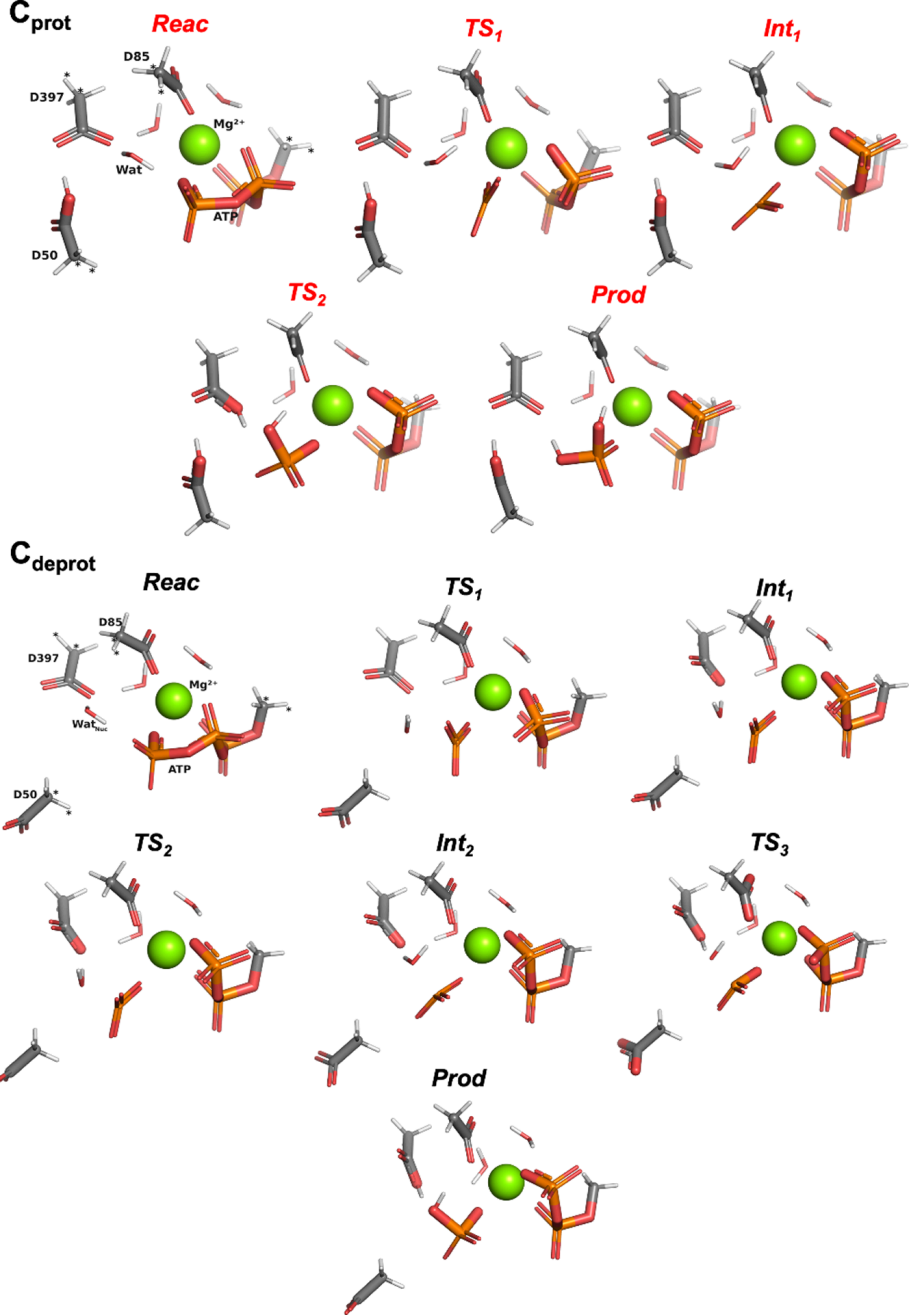
Stationary points (intermediates **Int**_**x**_ and first-order transition states **TS**_**x**_) encountered along the progress from **Reac** (cf. Figure 2) to **Prod** in cluster model **C**_**prot**_ (red; top) and cluster model **C**_**deprot**_ (black; bottom), optimized at the B3LYP/6-31G­(d) level. The color code is the same as in Figure 2. Bonds have been automatically drawn by the *PyMOL* suite^27^ based on interatomic distances. Atoms marked with “*” were kept frozen throughout the optimization process. All optimizations and final geometries are available online (see main text).

Hydrolysis in **C**_**prot**_ is, broadly speaking, reminiscent of the two-step process observed in **A**_**prot**_ and **B**_**prot**_. Like **Reac** in **B**_**prot**_, DFT optimization of **Reac** in **C**_**prot**_ also sees the proton shift spontaneously to Asp50; we therefore considered Asp397 as the base also in this case. Despite the switch in basic Asp, hydrolysis costs remain along the lines of those observed in **A**_**prot**_ (**TS**_**1**_ at +30.7 kcal mol^–1^ vs +31.0 kcal mol^–1^); this is likely because Wat_Nuc_ does not have to reorient itself to present one of its H to Asp397 as it does in **B**_**prot**_. **TS**_**1**_ and the resulting **Int**_**1**_ are structurally similar to the other two protonated cases (barring the change in base). What is interesting about this particular cluster model (Figure 6), and yet again suggests poorness of Asp397 as a base, is that when one of the Wat_Nuc_ protons is transferred to Asp397 and the system is optimized to generate the **Prod** state, the proton is promptly returned to another O of the inorganic phosphate, resulting in a complete [H_2_PO_4_]^−^. In other words, the **TS**_**2**_ separating **Int**_**1**_ and **Prod** does feature the Wat_Nuc_ proton closer to one of the Asp397:Oδ, but Asp397 merely acts as a facilitator of H^+^ between oxygens.

## Efficient Hydrolysis Requires Dyad Deprotonation

While we cannot be quantitative at this level of theory, a series of interesting observations emerge from the above data. Potential energy barriers (Δ*E*) remain high in all cases (over 20 kcal mol^–1^; Figure 3), as is to be expected with cluster models that are static and not dynamic, and that—in the case of the smaller models, for computational reasons—do not comprise some of the electronically beneficial elements of the active site such as the K^+^ cation and its coordinating residues (see Methods and Supporting Information). It is nonetheless clear from all six potential energy surface (PES) plots in Figure 3 that the rate-determining step, which is almost always the cleavage of ATP to P_i_ (except in **B**_**prot**_), consistently requires a lower potential energy barrier whenever the active site is deprotonated, regardless of dyad opening or closure. If we retrace the PES for the phosphate cleavage step in expanded versions of our six cluster models, reintroducing K^+^ and switching to a larger basis set (structures in Figure S3; energies in Figure S4; details in Methods), we find that this energetic trend is preserved along with the planar metaphosphate-like nature of the transition state.

Returning to plots in Figure 3, potential energy barrier differences (ΔΔ*E*) for rate-determining steps in clusters **A**_**deprot–prot**_, **B**_**deprot–prot**_, and **C**_**deprot–prot**_ are −7.0, −12.6, and −4.5 kcal mol^–1^, respectively, in favor of deprotonated models (−14.4, −16.2, and −0.5 kcal mol^–1^ in the larger versions; Figure S4). Together with proton transfer to one of the aspartates ceasing to be barrierless, and hydrolysis becoming more easily trapped in an ADP + [(H_2_O)­PO_3_]^−^ state (cf. **Int**_**1**_ in protonated models in Figures 4, 5, and 6), the higher hydrolysis costs in protonated models strongly suggest that the presence of the shared proton between Asp50 and Asp397 is evidently enough to dampen their basicity. More simply put, dyad protonation appears to switch off reactivity; in retrospect, this is consistent with starting CryoEM structures featuring a closed, likely protonated dyad and an unhydrolyzed ATP.

Upon further examination of the two series of ΔΔ*E* values reported in the previous paragraph, it is intriguing to note that energetic gains in **B**_**deprot**_ over **B**_**prot**_—where Wat_Nuc_ manages to stay lodged between both aspartates and the dyad remains closed (Figure 3b; Figure 5, **B**_**deprot**_, **Reac**)—are significantly greater than those observed in **A**_**deprot**_ vs **A**_**deprot**_ and **C**_**deprot**_ vs **C**_**deprot**_—where only one of Asp50 and Asp397 remains attached to Wat_Nuc_. However, we still have to note that, in the larger, B3LYP/6-311++G­(2d,2p)-optimized versions of the cluster models, hydrolysis in **A**_**deprot**_ with only Asp50 remaining attached to Wat_Nuc_ still costs less (+22.6 kcal mol^–1^) than hydrolysis in **B**_**deprot**_ (+29.8 kcal mol^–1^). Based on these data, it is therefore not directly possible to reconstruct how beneficial a closed dyad is to ATP hydrolysis compared to an open one since, for example, the lower cost in **A**_**deprot**_ could be due to other advantageously positioned elements of the active site.

Finally, our calculations also suggest that, perhaps in light of the higher number of hydrogen bonds formed by Asp397 that dissipate its negative charge, this always tends to act as a poorer base. This is reflected in higher hydrolysis costs whenever Asp397 is left to act as the base (**B**_**prot**_ and **C**_**deprot**_), or in its restitution of the proton in **C**_**prot**_.

## Conformational Variation and Deprotonation of the Catalytic Dyad

Overall, our data show that within **A**_**prot**_, **B**_**prot**_, **C**_**prot**_, **A**_**deprot**_, **B**_**deprot**_, and **C**_**deprot**_ there is already a microcosm of chemical information that can help us better elucidate some key aspects of the mechanism of ATP hydrolysis in Hsp60.

Indeed, even with as few as six cluster models, we did not find two identical cases. As we have seen, our evidence is sufficient to show that dyad protonation significantly hampers the basicity of both catalytic aspartates, raising the energetic cost of hydrolysis. There logically follows that in order to achieve a reaction-competent state, the proton must be lost. While “static” PropKa predictions^16^ mentioned at the start clearly point to a shared proton between Asp50 and Asp397 in the CryoEM structure of **D**, we have repeated p*K*_a_ predictions on the H++ server^28^ (output provided as Supporting Information), which takes into account side chain dynamics. Resulting Asp50/Asp397 p*K*_a_ predictions of 0.988/7.036 (H++ p*K*_1/2_ values) suggest that in a dynamic setting, the proton holding the unreactive dyad together can be lost after all. This is of course further corroborated by evidence from our previous MD simulations^13^ showing that, even when the dyad is protonated, the Asp50–Asp397 hydrogen bond can be broken on occasion, conformationally freeing the aspartates and making proton loss easier; possible candidates could include a nearby Asp393 that is within the reach of Asp50, or another active site H_2_O. Further evidence of Asp50/Asp397 side chain flexibility during MD simulations comes from a renewed analysis on active site flexibility carried out for this work (see Methods section and Supporting Information).

What happens once the dyad becomes deprotonated, on the other hand, and how it all relates to the pathogenic V72I mutation—which we recall is able to bring aspartates closer than in WT Hsp60 even when they are deprotonated^13^—is instead even more interesting to discuss. Here, two opposing forces are at work: on the one hand, the closer the dyad, the greater the basicity and potential benefit to hydrolysis; on the other hand, higher basicity equals of course higher proneness to protonate and, thus, to “self-extinguish” if the proton (or another positively charged species such as Na^+^) is recruited not from Wat_Nuc_ but from elsewhere.

Besides the contrasting trends in **A**_**deprot**_ and **B**_**deprot**_ emerging from our smaller vs larger cluster models, the other aspect that prevents us from automatically linking a closed and deprotonated dyad to more facile ATP hydrolysis is—as implied a few paragraphs earlier—not straightforwardly being able to quantify how readily a protonated dyad can lose its proton to become reactive and, conversely, how readily a deprotonated dyad that is reclosing can be quenched by an external proton/cation that is not coming from Wat_Nuc_. While our MD simulations^13^ cannot reproduce capture of isolated protons, they indeed suggest that deprotonated Asp50 and Asp397 are isolatedly able to recruit, e.g., stray Na^+^ countercations (which according to our models would leave one basic aspartate in play anyway), and this ability could increase as the enzyme forces two −1 charges closer together; we still assume that the best-placed protons to be recruited by the dyad are precisely those on Wat_Nuc_. We believe it is safe to hypothesize this even if, of course, through classical MD we are unable to verify the arrival of other protons from elsewhere.

## Role of the V72I Mutation

The difficulty we had in maintaining a closed dyad in our cluster models when they were deprotonated testifies just how energetically unfavorable it is to keep two negatively charged units close together. In light of this, the ability that Hsp60 has acquired to bring Asp50 and Asp397 together is quite a remarkable example of how sophisticatedly enzymes can evolve. It is all the more remarkable to consider that pathogenic mutation V72I distinctly brings Asp50 and Asp397 closer together even when they are deprotonated, as we proved in our previous classical MD investigation:^13^ while our cluster models were deliberately generated from simulations of WT and V72I Hsp60 alike (and did not feature the mutation site itself), the results presented in this work represent an additional confirmation that this may well be the molecular cause of altered ATPase activity in V72I Hsp60.

In conclusion, ATP hydrolysis is a deceivingly simple reaction that plays a crucial role in providing the necessary energy for chaperone proteins to perform their functions, ensuring proper orchestration of complex cellular processes. In the case of Hsp60, ATP binding induces conformational changes starting from the equatorial domain, which subsequently influence the intermediate and apical domains.

In this study, we extracted a series of independent, minimal cluster models of the Hsp60 active site from classical molecular dynamics simulations^13^ of its double ring assembly **D** and proceeded to map the potential energy surface for ATP hydrolysis in each one, employing density functional theory, and with the aim of identifying a plausible reactivity scenario. In building our models, we focused exclusively on the molecular actors involved. Our main focus was on the role of a catalytic active site dyad composed of aspartates Asp50 and Asp397, which Hsp60 is able to bring close together even without a shared proton between the two that dampens the total charge of −2 (and pathogenic V72I Hsp60 even more so).

Our results eloquently show that whenever a shared proton is present between the catalytic aspartates, energetic costs for ATP hydrolysis rise considerably. Conversely, in deprotonated cluster models, even when the accumulation of negative charge drives the aspartates away and only one of the two remains as the base, energetic costs remain low. All of these findings suggest that reactivity in Hsp60, modestly enhanced in its V72I mutant, is associated with a deprotonated catalytic dyad.

To the best of our knowledge, this is the first study systematically exploring the reactivity of Hsp60. Our findings contribute to the broader understanding of the molecular mechanisms governing this complex chaperonin and provide a key to rationalize any variation in them whenever an external perturbation arises (e.g., a mutation or an allosteric ligand).

## Methods

Distinct p*K*_a_ predictions for Asp50 and Asp397 in the starting CryoEM structure of **D** (PDB: 8G7L)^10^ were obtained using PropKa (version 3.1)^16^ and the H++ server.^28^

Protonated cluster models **A**_**prot**_, **B**_**prot**_, and **C**_**prot**_ were excided from classical MD simulations of 14-meric complex **D** ^13^ (Table 1), after choosing them based on the structural and electronic criteria discussed above and as Supporting Information.

Our main methodological references for this work are previous cluster model studies by the Himo group (e.g., refs 17 and 18). We did, in fact, introduce some simplifications to the standard protocol. More specifically, the only elements we chose to include in the simplified cluster models were catalytic dyad side chains, a fully hexacoordinated Mg^2+^ (3 ATP oxygens; 2 H_2_O; Asp85), Wat_Nuc_, and the triphosphate portion of ATP with a terminal methyl replacing the C–C bond to the ribose moiety. Asp side chains were cut at the Cβ–Cα bond rather than the customary Cα–N and Cα–C (with Cα replaced by a third hydrogen). This simplification allowed us to reduce the number of electrons and degrees of freedom and to circumvent problems related to the inclusion of the K^+^ coordination sphere and other electronically relevant fragments. However, it came at the cost of removing some of the actors that typically stabilize the substrates by withdrawing negative charge (cf. Supporting Information). Atoms that were kept frozen in each model were: Cβ and the Hβ replacing Cα on Asp50, Asp85, and Asp397; and the remaining C and one of the methyl H on ATP. All are marked with “*” in models illustrated in Figure 2.

We mention that this simplification does not impact model flexibility. As Supporting Information (Figure S5), we have superimposed all 28 optimized stationary points found for **A**_**prot**_, **B**_**prot**_, **C**_**prot**_, **A**_**deprot**_, **B**_**deprot**_, and **C**_**deprot**_ (Figures 4–6) onto the parent MD poses from which **A**_**prot**_, **B**_**prot**_, and **C**_**prot**_ were first excided (Table 1; Figure S1): it is clear that optimization gives all side chains ample room to relax toward different directions of the active site cavity, and that in deprotonated models, negatively charged Asp50 and Asp397 side chains are sufficiently free to distance themselves. It is furthermore encouraging to note that no atom in any of the stationary points is ever found to clash with Hsp60 atoms from parent MD poses excluded from the cluster models. Further on the issue of flexibility, we have analyzed the distributions of the 15 Cα–Cα distances associated with the six residues present in the enlarged versions of our cluster models (see details below and the aforementioned Figure S3) during our previously published MD simulations of WT and V72I **D** in three protonation states.^13^ We plot these 90 distributions in Figures S6–S20, and overlay on each panel the value of the corresponding Cα–Cα distance in the MD pose from which **A**_**prot**_, **B**_**prot**_, and **C**_**prot**_ were excided: on top of showing that our MD simulations capture ample active site flexibility in all protonation states, plots indicate that even within the constraint of having to ensure “reactive” catalytic distances, our cluster models are sufficiently representative of this flexibility.

Returning to the smaller cluster models, starting from **A**_**prot**_, **B**_**prot**_, and **C**_**prot**_ in their **Reac** state, all stationary points for all cluster models on the path to ATP hydrolysis were located and optimized using the *Gaussian16* package,^29^ employing Density Functional Theory (DFT) at the B3LYP/6-31G­(d) level.^19,20^ To implicitly account for the effects of the protein surroundings, as prescribed by our reference studies,^18,30^ we applied the (implicit) Solvation Model Density (SMD) approach^31^ using water as the nominal implicit solvent, but with the dielectric constant fictitiously set to 4. Deprotonated cluster models **A**_**deprot**_, **B**_**deprot**_, and **C**_**deprot**_ were generated by deprotonating one or more of the stationary points from their protonated counterparts, always considering the **Reac** state and, if deemed necessary, one of the other stationary points. Following deprotonation, we always reoptimized at the same level of DFT.

In particular, in the case of **A**_**deprot**_, it was sufficient to generate only the **Reac** state, and in the case of **C**_**deprot**_, we also generated what became **Int**_**2**_ by deprotonating **Int**_**1**_ of **C**_**prot**_. For **B**_**deprot**_, **Prod** arose directly after optimizing the deprotonated **Int**_**1**_ state of **B**_**prot**_, which immediately led to barrierless proton transfer to Asp50. Reactive state **Reac**, which featured a deprotonated but closed catalytic dyad (Figure 2), was achieved by reconstructing the ATP molecule, moving the Pγ atom toward Oβ only. The atoms composing Wat_Nuc_ were left untouched and, when optimized, spontaneously re-formed the full Wat_Nuc_ visible in Figure 2.

Broadly speaking, the strategy to locate stationary points along the PES was as follows for all six cluster models: first, the estimated **Prod** state was generated manually by moving as few atoms as possible: Pγ and Wat_Nuc_:O to create a new bond and one of the Wat_Nuc_:H to its closest Asp50 and Asp397 Oδ. The **Prod** state was then optimized, and there systematically followed a series of Synchronous Transit-Guided Quasi-Newton (STQN) calculations^32^ and Intrinsic Reaction Coordinate (IRC) calculations^33^ (with the GS2 algorithm^34^ if the standard one did not converge) to progressively locate all stationary points separating **Reac** and **Prod**. Specifically, STQN calculations were used to locate a transition state **TS**_**x**_ separating two input minima and were automatically followed by IRC calculations to verify that each **TS**_**x**_ fell back to each of the input minima. If this did not occur and a new minimum or minima were found, a new round of STQN + IRC was repeated with the new minimum/minima as input. This was repeated until a continuous path was obtained linking **Reac** and **Prod**.

To locate **TS**_**3**_ in **C**_**deprot**_, we first performed a constrained optimization on an input structure with the proton halfway between Asp397:Oδ and former Wat_Nuc_:O and used the output geometry in an unconstrained DFT optimization to locate a first-order saddle point.

Frequency calculations were carried out on all stationary points to confirm their nature as minima (all positive vibrational frequencies) or first-order TSs (one negative vibrational frequency).

To check that geometries and qualitative energetic trends were invariant to basis set and cluster model size—particularly in light of the aforementioned problems linked to omission of K^+^—we reoptimized potential energy surfaces for the sole phosphate cleavage in enlarged versions of all six cluster models, this time coupling the B3LYP functional with the larger triple-ζ 6-311++G­(2d,2p) basis set. Extra atoms excided from “parent” MD poses in Figure S1 and Table 1 include the (henceforth frozen) K^+^, side chains of Thr28 and Thr88 coordinating K^+^ (up to Cβ), and side chain of Thr87 hydrogen-bonded to ATP:Oγ (up to Cβ). Like in the smaller models, Cβ and the Hβ replacing Cα on the newly added Thr residues were frozen alongside K^+^. To save computational time, IRC calculations were not carried out, and the extra excided atoms were directly pasted into the already-optimized cluster models (but were themselves subjected to reoptimization). All stationary points are shown in Figure S3, while energies are plotted in Figure S4.

## Supplementary Material





## Data Availability

All calculations are available electronically online (Link: 10.19061/iochem-bd-6-535).^26^

## Notes

Catalytically relevant features of the Hsp60 active site, selection of “reactive” poses, parent MD poses from which cluster models were excided, similar MD poses in MD simulations of the corresponding Hsp60 variant, findings with larger cluster models, tests on cluster model flexibility (PDF)
H++ output (ZIP)

## References

[1] Bukau B., Horwich A. L. (1998). The Hsp70 and Hsp60
Chaperone Machines. Cell.

[2] Cheng M. Y., Hartl F. U., Martin J., Pollock R. A., Kalousek F., Neuper W., Hallberg E. M., Hallberg R. L., Horwich A. L. (1989). Mitochondrial
Heat-Shock Protein Hsp60 Is Essential for Assembly of Proteins Imported
into Yeast Mitochondria. Nature.

[3] Kim Y. E., Hipp M. S., Bracher A., Hayer-Hartl M., Hartl F. U. (2013). Molecular Chaperone Functions in
Protein Folding and
Proteostasis. Annu. Rev. Biochem..

[4] Zuo W.-F., Pang Q., Zhu X., Yang Q.-Q., Zhao Q., He G., Han B., Huang W. (2024). Heat shock proteins as hallmarks
of cancer: insights from molecular mechanisms to therapeutic strategies. Journal of Hematology & Oncology.

[5] Ghosh J. C., Dohi T., Kang B. H., Altieri D. C. (2008). Hsp60 Regulation
of Tumor Cell Apoptosis*. J. Biol. Chem..

[6] Lassila J. K., Zalatan J. G., Herschlag D. (2011). Biological Phosphoryl-Transfer Reactions:
Understanding Mechanism and Catalysis. Annu.
Rev. Biochem..

[7] Kamerlin S. C. L., Sharma P. K., Prasad R. B., Warshel A. (2013). Why nature really chose
phosphate. Q. Rev. Biophys..

[8] Gomez-Llorente Y., Jebara F., Patra M., Malik R., Nisemblat S., Chomsky-Hecht O., Parnas A., Azem A., Hirsch J. A., Ubarretxena-Belandia I. (2020). Structural
basis for active single and double ring
complexes in human mitochondrial Hsp60-Hsp10 chaperonin. Nat. Commun..

[9] Wang J. C.-Y., Chen L. (2021). Structural basis for the structural
dynamics of human
mitochondrial chaperonin mHsp60. Sci. Rep..

[10] Braxton J. R., Shao H., Tse E., Gestwicki J. E., Southworth D. R. (2024). Asymmetric apical domain states of mitochondrial Hsp60
coordinate substrate engagement and chaperonin assembly. Nature Structural & Molecular Biology.

[11] Koike-Takeshita A., Mitsuoka K., Taguchi H. (2014). Asp-52 in
Combination with Asp-398
Plays a Critical Role in ATP Hydrolysis of Chaperonin GroEL*. J. Biol. Chem..

[12] Brocchieri L., Karlin S. (2000). Conservation among
HSP60 sequences in relation to structure,
function, and evolution. Protein Sci..

[13] Torielli L., Guarra F., Shao H., Gestwicki J. E., Serapian S. A., Colombo G. (2025). Pathogenic mutation
impairs functional
dynamics of Hsp60 in mono- and oligomeric states. Nat. Commun..

[14] Syed A., Zhai J., Guo B., Zhao Y., Wang J. C.-Y., Chen L. (2024). Cryo-EM structure and molecular dynamic simulations
explain the enhanced stability and ATP activity of the pathological
chaperonin mutant. Structure.

[15] Chen L., Syed A., Balaji A. (2022). Hereditary
spastic paraplegia SPG13
mutation increases structural stability and ATPase activity of human
mitochondrial chaperonin. Sci. Rep..

[16] Olsson M. H. M., Søndergaard C. R., Rostkowski M., Jensen J. H. (2011). PROPKA3: Consistent Treatment of
Internal and Surface
Residues in Empirical pK_a_ Predictions. J. Chem. Theory Comput..

[17] Sheng X., Himo F. (2023). The Quantum Chemical
Cluster Approach in Biocatalysis. Acc. Chem.
Res..

[18] Planas F., McLeish M. J., Himo F. (2019). Computational Study
of Enantioselective
Carboligation Catalyzed by Benzoylformate Decarboxylase. ACS Catal..

[19] Becke A. D. (1993). Density-functional
thermochemistry. III. The role of exact exchange. J. Chem. Phys..

[20] Lee C. T., Yang W. T., Parr R. G. (1988). Development
of the Colle-Salvetti
Correlation-Energy Formula into a Functional of the Electron-Density. Phys. Rev. B.

[21] Serapian S. A., van der Kamp M. W. (2019). Unpicking the Cause of Stereoselectivity in Actinorhodin
Ketoreductase Variants with Atomistic Simulations. ACS Catal..

[22] Serapian S. A., Crosby J., Crump M. P., van der Kamp M. W. (2022). Path to
Actinorhodin: Regio- and Stereoselective Ketone Reduction by a Type
II Polyketide Ketoreductase Revealed in Atomistic Detail. JACS Au.

[23] Todd M. J., Viitanen P. V., Lorimer G. H. (1993). Hydrolysis of adenosine 5′-triphosphate
by Escherichia coli GroEL: Effects of GroES and potassium ion. Biochemistry.

[24] Rye H. S., Burston S. G., Fenton W. A., Beechem J. M., Xu Z., Sigler P. B., Horwich A. L. (1997). Distinct
actions of cis and trans
ATP within the double ring of the chaperonin GroEL. Nature.

[25] Serapian S. A., Moroni E., Ferraro M., Colombo G. (2021). Atomistic Simulations
of the Mechanisms of the Poorly Catalytic Mitochondrial Chaperone
Trap1: Insights into the Effects of Structural Asymmetry on Reactivity. ACS Catal..

[26] Álvarez-Moreno M., de Graaf C., López N., Maseras F., Poblet J. M., Bo C. (2015). Managing the
Computational Chemistry Big Data Problem: The ioChem-BD
Platform. J. Chem. Inf. Model..

[27] DeLano, W. L. The PyMOL Molecular Graphics System, Version 2.4.0a0; Schrödinger LLC: New York, NY, U.S.A., 2021.

[28] Gordon J. C., Myers J. B., Folta T., Shoja V., Heath L. S., Onufriev A. (2005). H++: a server for estimating p Ka s and adding missing
hydrogens to macromolecules. Nucleic Acids Res..

[29] Frisch, M. J. ; Trucks, G. W. ; Schlegel, H. B. ; Scuseria, G. E. ; Robb, M. A. ; Cheeseman, J. R. ; Scalmani, G. ; Barone, V. ; Petersson, G. A. ; Nakatsuji, H. ; Li, X. ; Caricato, M. ; Marenich, A. V. ; Bloino, J. ; Janesko, B. G. ; Gomperts, R. ; Mennucci, B. ; Hratchian, H. P. ; Ortiz, J. V. ; Izmaylov, A. F. ; Sonnenberg, J. L. ; Williams-Young, D. ; Ding, F. ; Lipparini, F. ; Egidi, F. ; Goings, J. ; Peng, B. ; Petrone, A. ; Henderson, T. ; Ranasinghe, D. ; Zakrzewski, V. G. ; Gao, J. ; Rega, N. ; Zheng, G. ; Liang, W. ; Hada, M. ; Ehara, M. ; Toyota, K. ; Fukuda, R. ; Hasegawa, J. ; Ishida, M. ; Nakajima, T. ; Honda, Y. ; Kitao, O. ; Nakai, H. ; Vreven, T. ; Throssell, K. ; Montgomery, J. A., Jr. ; Peralta, J. E. ; Ogliaro, F. ; Bearpark, M. J. ; Heyd, J. J. ; Brothers, E. N. ; Kudin, K. N. ; Staroverov, V. N. ; Keith, T. A. ; Kobayashi, R. ; Normand, J. ; Raghavachari, K. ; Rendell, A. P. ; Burant, J. C. ; Iyengar, S. S. ; Tomasi, J. ; Cossi, M. ; Millam, J. M. ; Klene, M. ; Adamo, C. ; Cammi, R. ; Ochterski, J. W. ; Martin, R. L. ; Morokuma, K. ; Farkas, O. ; Foresman, J. B. ; Fox, D. J. ; Gaussian 16 Rev. C.02; Gaussian, Inc.; Wallingford, CT, U.S.A., 2016.

[30] Himo F., de Visser S. P. (2022). Status
report on the quantum chemical cluster approach
for modeling enzyme reactions. Communications
Chemistry.

[31] Marenich A. V., Cramer C. J., Truhlar D. G. (2009). Universal Solvation Model Based on
Solute Electron Density and on a Continuum Model of the Solvent Defined
by the Bulk Dielectric Constant and Atomic Surface Tensions. J. Phys. Chem. B.

[32] Peng C., Bernhard Schlegel H. (1993). Combining
Synchronous Transit and Quasi-Newton Methods
to Find Transition States. Isr. J. Chem..

[33] Fukui K. (1981). The path of
chemical reactions - the IRC approach. Acc.
Chem. Res..

[34] Gonzalez C., Schlegel H. B. (1990). Reaction path following in mass-weighted internal coordinates. J. Phys. Chem..

