# Supplementary material for: Density Functional Theory Calculations to Investigate the Role Played by an Aspartate Dyad in Hsp60-Catalyzed ATP Hydrolysis
Source: J Phys Chem Lett. 2025 Oct 10;16(42):10879–90. doi: 10.1021/acs.jpclett.5c02351 (PMC12557358; doi:10.1021/acs.jpclett.5c02351)
Supplement: Supplementary file 1 [file jz5c02351_si_001.pdf]

SUPPORTING INFORMATION

Density Functional Theory calculations to investigate the role played by an aspartate dyad in Hsp60-catalysed ATP hydrolysis

Luca Torielli, Federica Guarra, Stefano A. Serapian,* Giorgio Colombo*

Department of Chemistry, University of Pavia, via Taramelli 12, 27100 Pavia, Italy

*Authors to whom correspondence should be addressed:
Stefano A. Serapian: stefanoartin.serapian@unipv.it
Giorgio Colombo: g.colombo@unipv.it

Catalytically relevant features of the Hsp60 active site and selection of "reactive" poses

The Hsp60 active site features several electronically important actors besides the catalytic Asp dyad. These include: (1) a Mg^{2+} cation to which ATP chelates with three oxygens (one each α, β, γ), and which must also be coordinated to two H_2O molecules and to Asp85; (2) a K^+ cation, which withdraws negative charge from the transition state, together with its two coordinating H_2O molecules and coordinating residues Thr28, Thr88 and Lys49. When isolating suitable poses from our MD simulations, of course, we therefore had to make sure that the above interactions were retained, *i.e.*, that the conformation in the reactive site was functional to react.

To systematically identify the most catalytically competent conformations arising in each monomer, we performed a "reactive pose" analysis focusing on the active site geometry. Specifically, we evaluated key distances and angles among the most functionally critical residues involved in ATP hydrolysis. These were as follows:

1) ATP:Pγ – Wat_{Nuc}:O distance no greater than 2.45 Å

2) Wat_{Nuc}:O – ATP:Pγ – ATP:Oβ attack angle above 135°

3) Mg^{2+} coordination:

   a) Asp85:Oδ – Mg^{2+} distance lower than 2.45 Å

   b) Two Wat:O – Mg^{2+} distances both lower than 2.4 Å

   c) ATP:Oα – Mg^{2+} distance lower than 2.25 Å

   d) ATP:Oβ – Mg^{2+} distance lower than 2.25 Å

   e) ATP:Oγ – Mg^{2+} distance lower than 2.2 Å

4) K^+ coordination:

   a) Thr28:Oγ1 – K^+ distance lower than 3.4 Å

   b) Thr88:Oγ1 – K^+ distance lower than 3.5 Å

   c) Thr28:O – K^+ distance lower than 4 Å

   d) Lys49:O – K^+ distance lower than 3.1 Å

   e) Two Wat:O – K^+ distances both lower than 3.75 Å

   f) ATP:Oα – K^+ distance lower than 3.05 Å

   g) ATP:Oγ – K^+ distance lower than 3.2 Å

5) Asp50:Cγ – Asp397:Cγ distance lower than 6.4 Å

6) ATP:Oγ – Thr87:Hγ1 distance lower than 2.2 Å

By applying these rational geometric thresholds, we were able to classify conformations as "reactive".

MD Poses from which cluster models were isolated

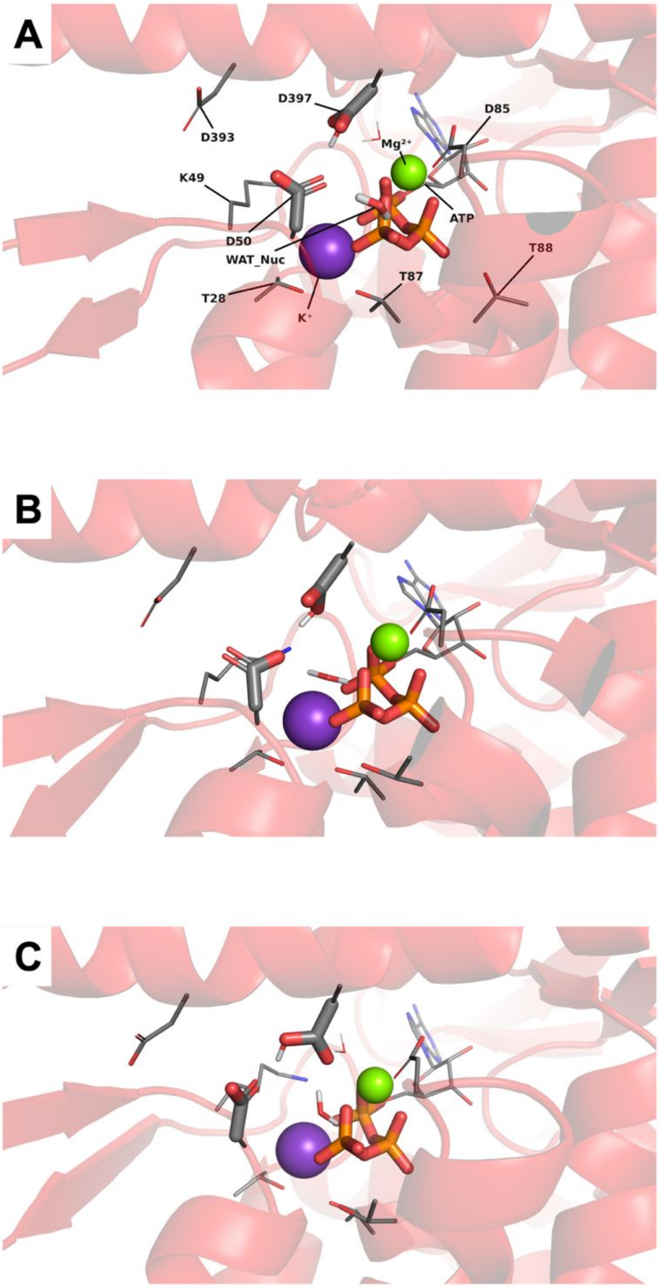

Figure S1. Snapshots of the active site of three different Hsp60 monomers, extracted from across our MD simulations of 14-meric complex **D** as *per* the details provided in Table 1 of the main text. These were the original MD poses from which we derived cluster models $\mathbf{A_{prot}}$, $\mathbf{B_{prot}}$, and $\mathbf{C_{prot}}$, and optimised them at the B3LYP/6-31G(*d*) level to obtain **Reac**. Colour code is identical to Figure 1b in the main text.

Similar poses from MD Simulations of Hsp60 variants

Table S1. Two classical MD simulation poses of **D** with catalytic dyad arrangements similar to those chosen from another variant to initially generate cluster models $\mathbf{A_{prot}}$ to $\mathbf{C_{prot}}$. These same poses are represented in FigureS2.

Similar to pose(s) generating model(s)	***Replica (/4)***	***Frame (/10000)***	***Protomer (/14)***	***Variant***
$\mathbf{A_{prot}}$ & $\mathbf{B_{prot}}$	1	1293	3	WT
$\mathbf{C_{prot}}$	1	3841	2	V72I

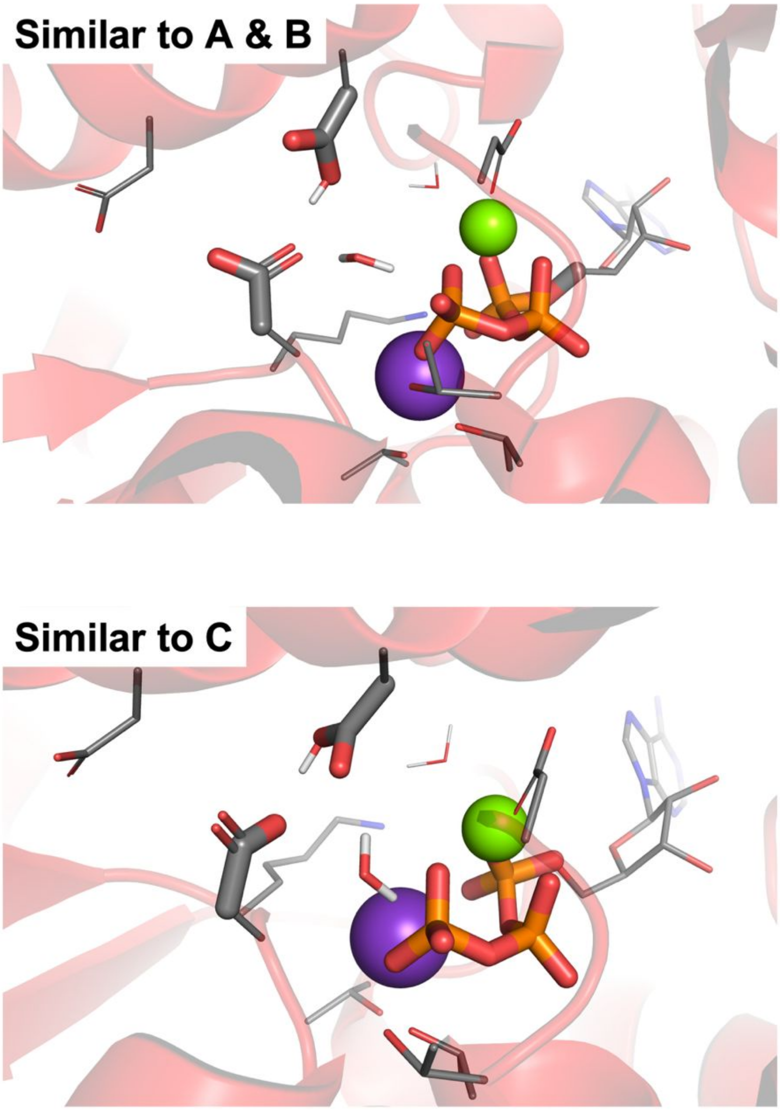

Figure S2. Snapshots of the active site of two different Hsp60 monomers, extracted from across our MD simulations of 14-meric complex **D**, as *per* the details provided in Table S1 above. In the top panel, the pose is similar to poses A and B in Figure S1, but comes from MD simulations of WT **D**. In the bottom panel, the pose is similar to pose C in Figure S1, but comes from MD simulations of V72I **D**. The colour code is identical to Figure 1b in the main text. Represented residues are the same as those in Figure S1.

Phosphate cleavage at higher levels of theory and with larger models

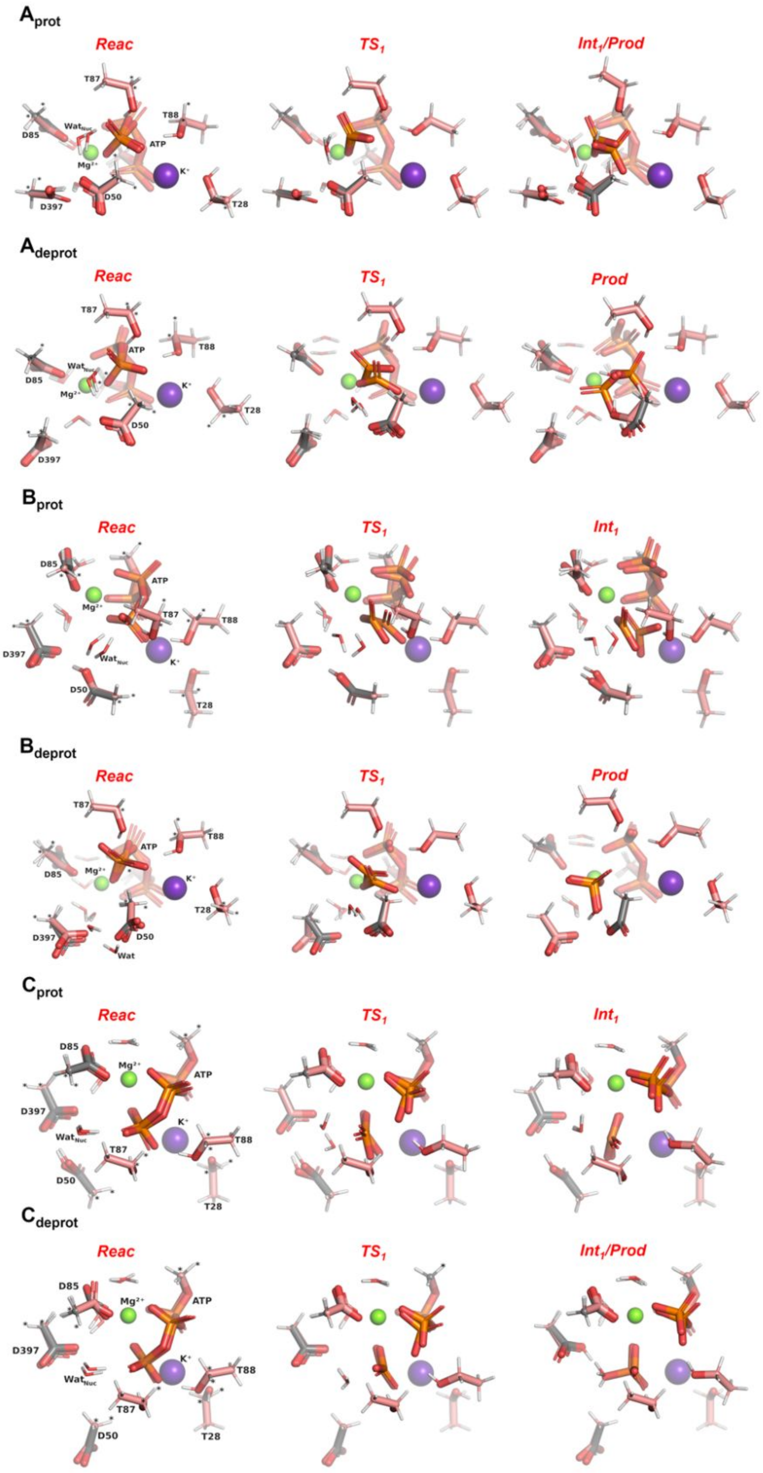

Figure S3. Structural comparison of the first three stationary points (**Reac**; $\mathbf{TS_1}$; $\mathbf{Int_1}$ or **Prod** depending on proton transfer) encountered during the phosphate cleavage step of ATP hydrolysis in cluster models $\mathbf{A_{prot}}$, $\mathbf{B_{prot}}$, $\mathbf{C_{prot}}$, $\mathbf{A_{deprot}}$, $\mathbf{B_{deprot}}$, and $\mathbf{C_{deprot}}$, optimised at the B3LYP/6-31G(*d*) level (*cf.* Figures 4 to 6; C atoms in grey); and in their enlarged versions, optimised at the B3LYP/6-311++G(*2d*,*2p*) level (C atoms in salmon). Cluster models are superimposed at common immobilised Cα atoms. All residues and cations are labelled on the leftmost panels; threonines and K^+ are only featured in the enlarged models (see main text).

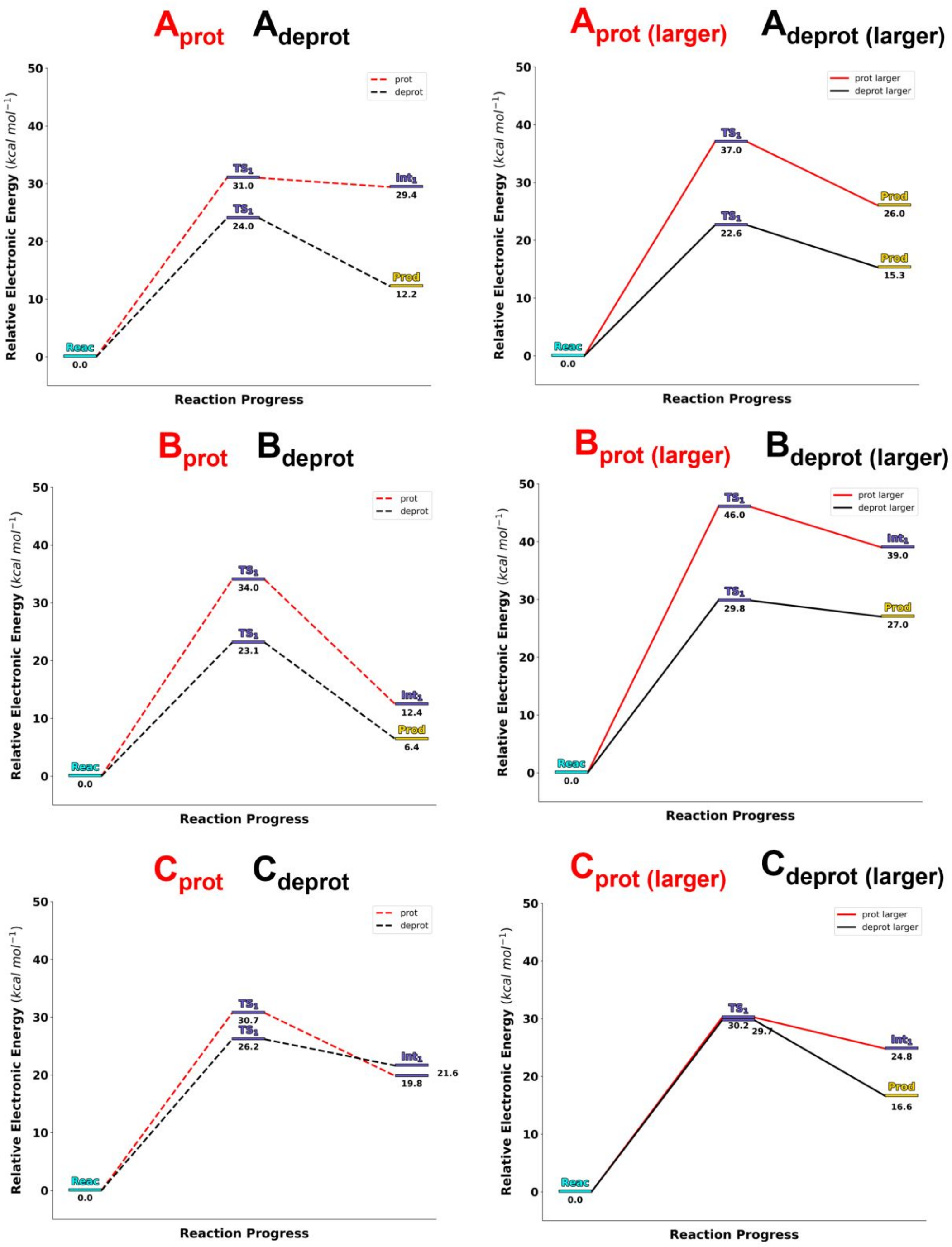

Figure S4. Comparison of electronic energy profiles for the sole phosphate cleavage step in (left) original cluster models $\mathbf{A}_{\mathbf{prot}}$, $\mathbf{B}_{\mathbf{prot}}$, $\mathbf{C}_{\mathbf{prot}}$, $\mathbf{A}_{\mathbf{deprot}}$, $\mathbf{B}_{\mathbf{deprot}}$, and $\mathbf{C}_{\mathbf{deprot}}$ (*cf.* Figures 3 to 6; optimised at the B3LYP/6-31G(*d*) level) *vs.* (right) their enlargements with K^+ and three additional threonines (Figure S3; details in main text; optimised at the B3LYP/6-311++G(*2d*,*2p*) level). Protonated profiles are in red, deprotonated profiles in black. Scales are identical, but compared to Figure 3, stationary points **Prod** and $\mathbf{Int_1}$ have been brought at the same level to facilitate visual comparison. Note that some $\mathbf{Int_1}$ directly evolve into **Prod** when enlarging cluster model and basis set.

Cluster model flexibility

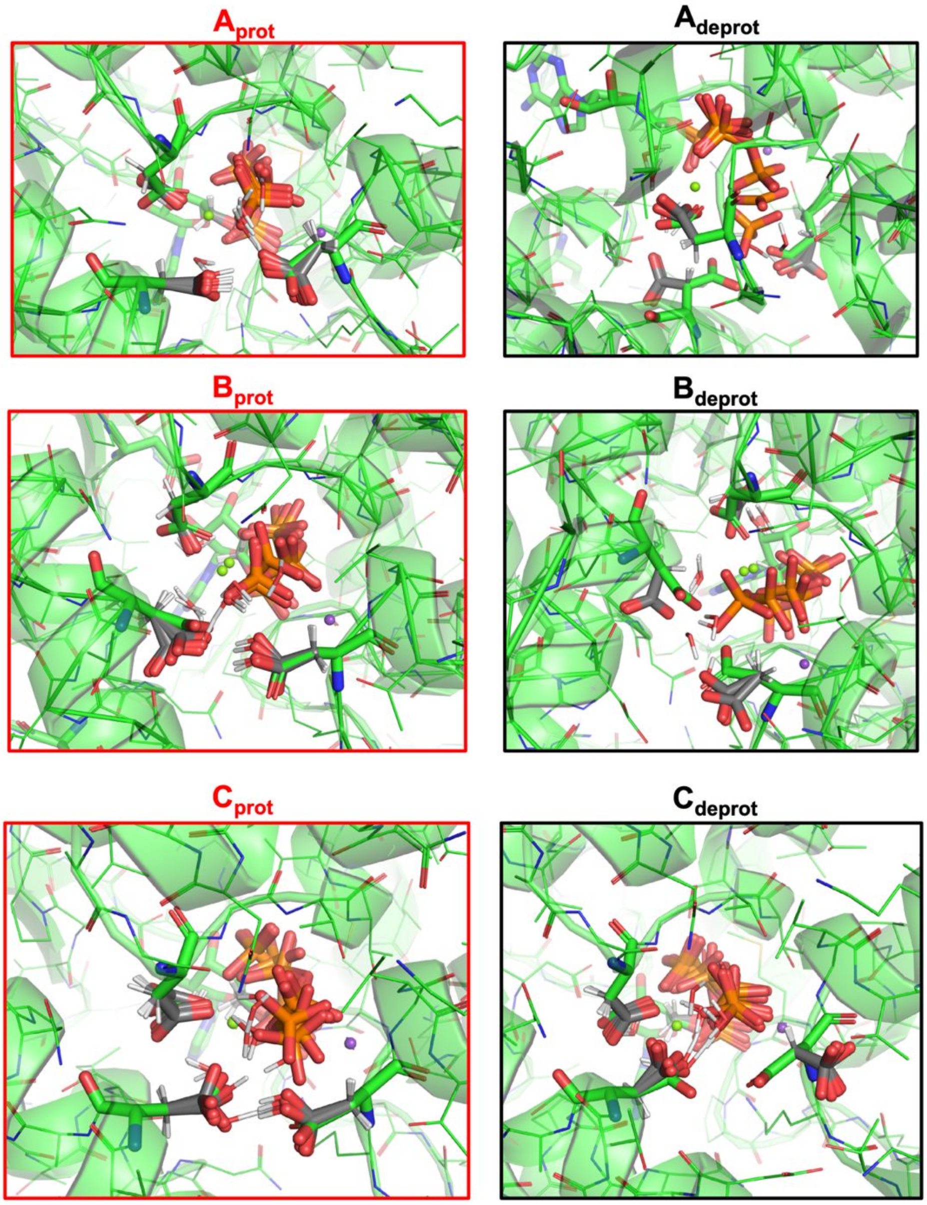

Figure S5. Superposition on parent MD poses A to C (*cf.* Table 1; Figure S1) of all stationary points found while mapping the potential energy surface of models $\mathbf{A}_{prot}$ to $\mathbf{C}_{prot}$ (top left to bottom left); and models $\mathbf{A}_{deprot}$ to $\mathbf{C}_{deprot}$ (top right to bottom right). These include reactants, products, intermediates, and first-order transition states. Original MD poses are rendered as green cartoons, with C atoms as green lines or sticks (if belonging to a residue partly transferred to cluster models). Cluster model stationary points have their atoms rendered as sticks or spheres, with C atoms rendered in grey. Colour code for remaining atoms: purple: K; green (sphere): Mg; orange: P; red: O; blue: N; off-white: H. Non-cluster model hydrogen atoms omitted for clarity.

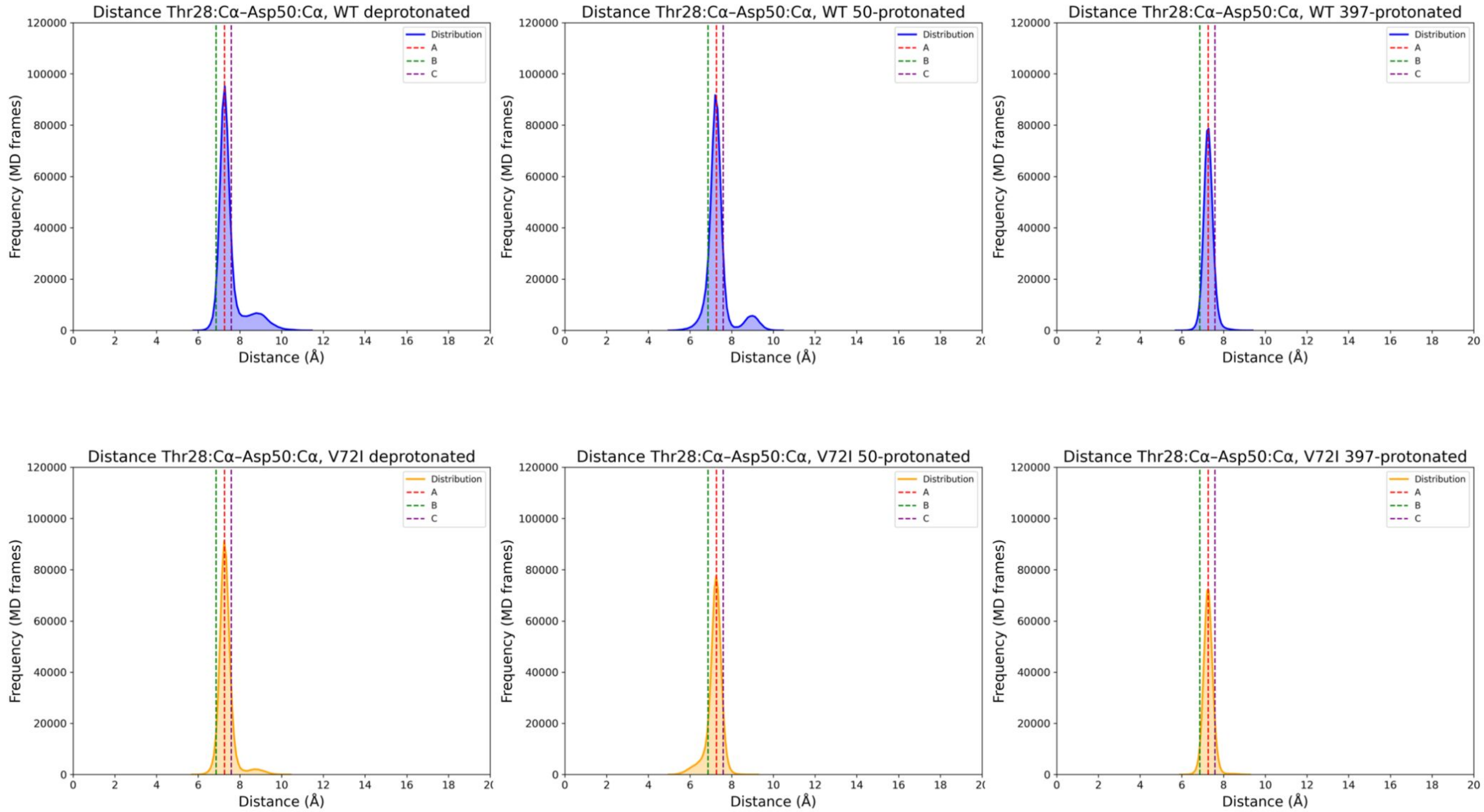

Figure S6. Histograms showing the distribution of the Thr28:Cα–Asp50:Cα distance during the course of our previously published MD simulations (L. Torielli *et al.*, *Nat. Commun.* **2025**, *16*, 3158) of: WT **D** (top; blue histograms); and V72I **D** (bottom; orange histograms); with: Asp50 and Asp397 both deprotonated (leftmost panels); Asp50 protonated (central panels); or Asp397 protonated (rightmost panels). Overlaid dotted lines denote the value of the same Cα–Cα distance in the MD frame from which we excided cluster models **A**$_{\mathbf{prot}}$, **B**$_{\mathbf{prot}}$, **C**$_{\mathbf{prot}}$.

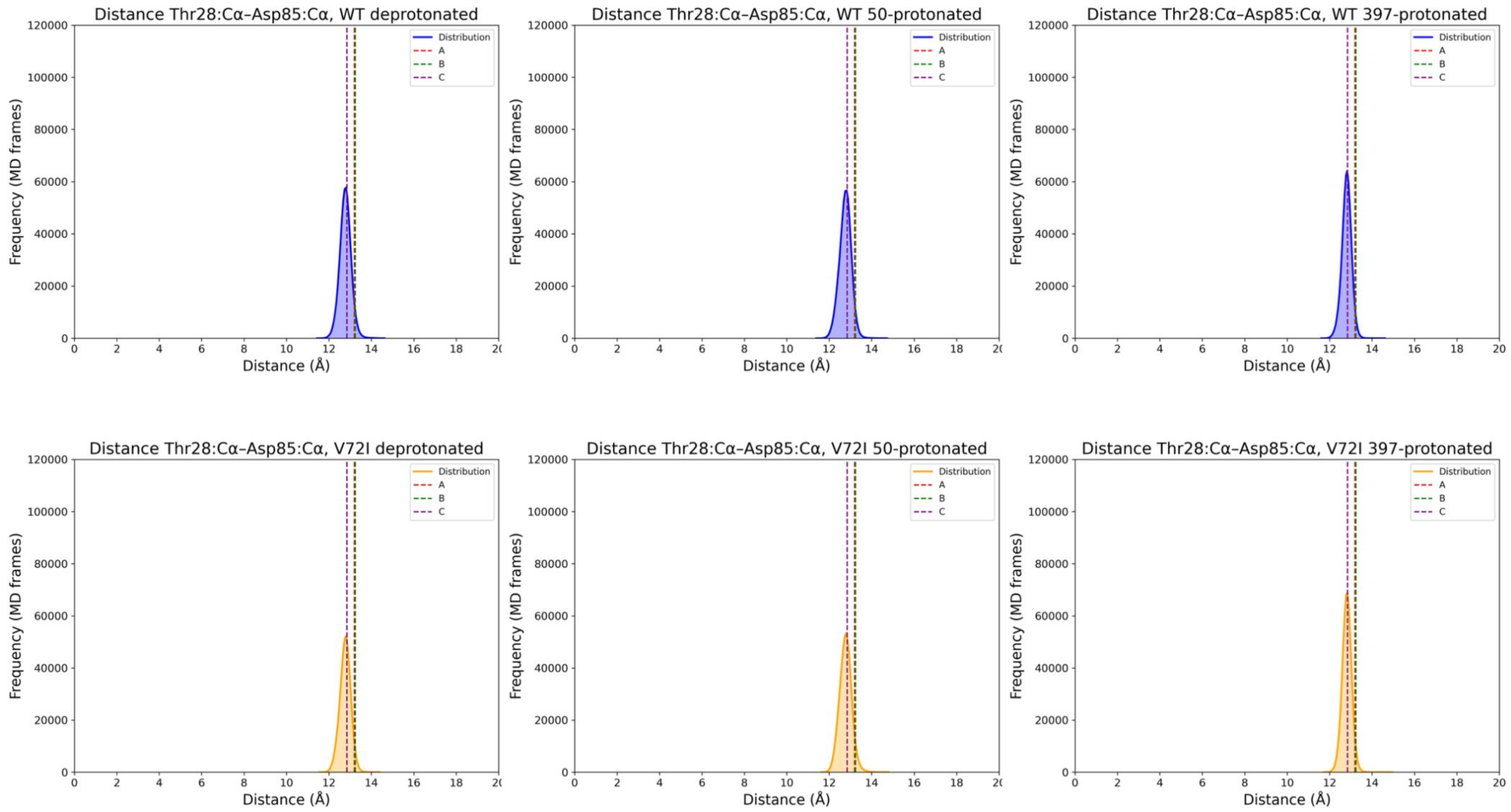

Figure S7. Histograms showing the distribution of the Thr28:Cα–Asp85:Cα distance during the course of our previously published MD simulations (L. Torielli *et al.*, *Nat. Commun.* **2025**, *16*, 3158) of: WT **D** (top; blue histograms); and V72I **D** (bottom; orange histograms); with: Asp50 and Asp397 both deprotonated (leftmost panels); Asp50 protonated (central panels); or Asp397 protonated (rightmost panels). Overlaid dotted lines denote the value of the same Cα–Cα distance in the MD frame from which we excided cluster models $\mathbf{A_{prot}}$, $\mathbf{B_{prot}}$, $\mathbf{C_{prot}}$.

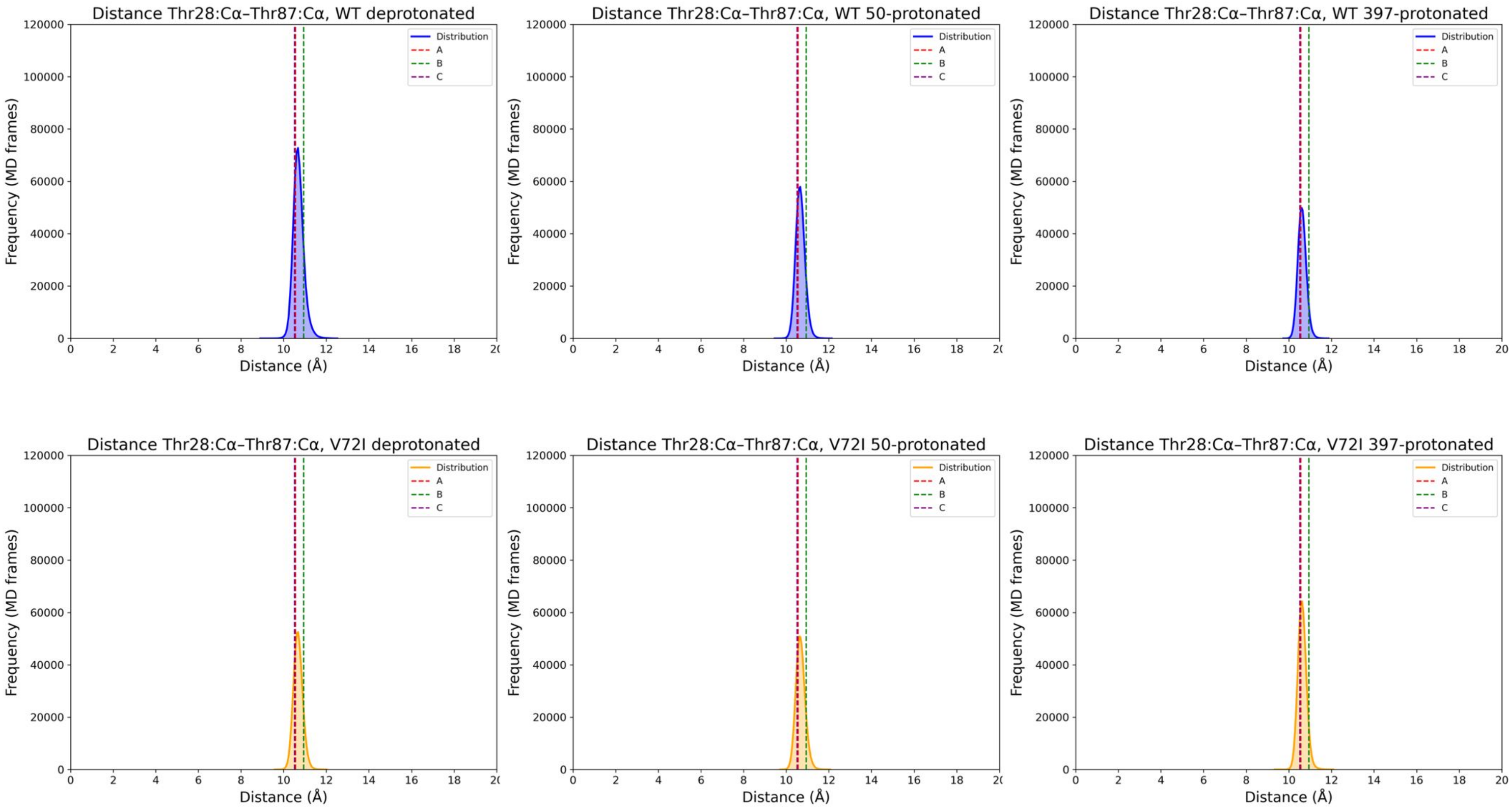

Figure S8. Histograms showing the distribution of the Thr28:Cα–Thr87:Cα distance during the course of our previously published MD simulations (L. Torielli *et al.*, *Nat. Commun.* **2025**, *16*, 3158) of: WT **D** (top; blue histograms); and V72I **D** (bottom; orange histograms); with: Asp50 and Asp397 both deprotonated (leftmost panels); Asp50 protonated (central panels); or Asp397 protonated (rightmost panels). Overlaid dotted lines denote the value of the same Cα–Cα distance in the MD frame from which we excided cluster models $\mathbf{A_{prot}}$, $\mathbf{B_{prot}}$, $\mathbf{C_{prot}}$.

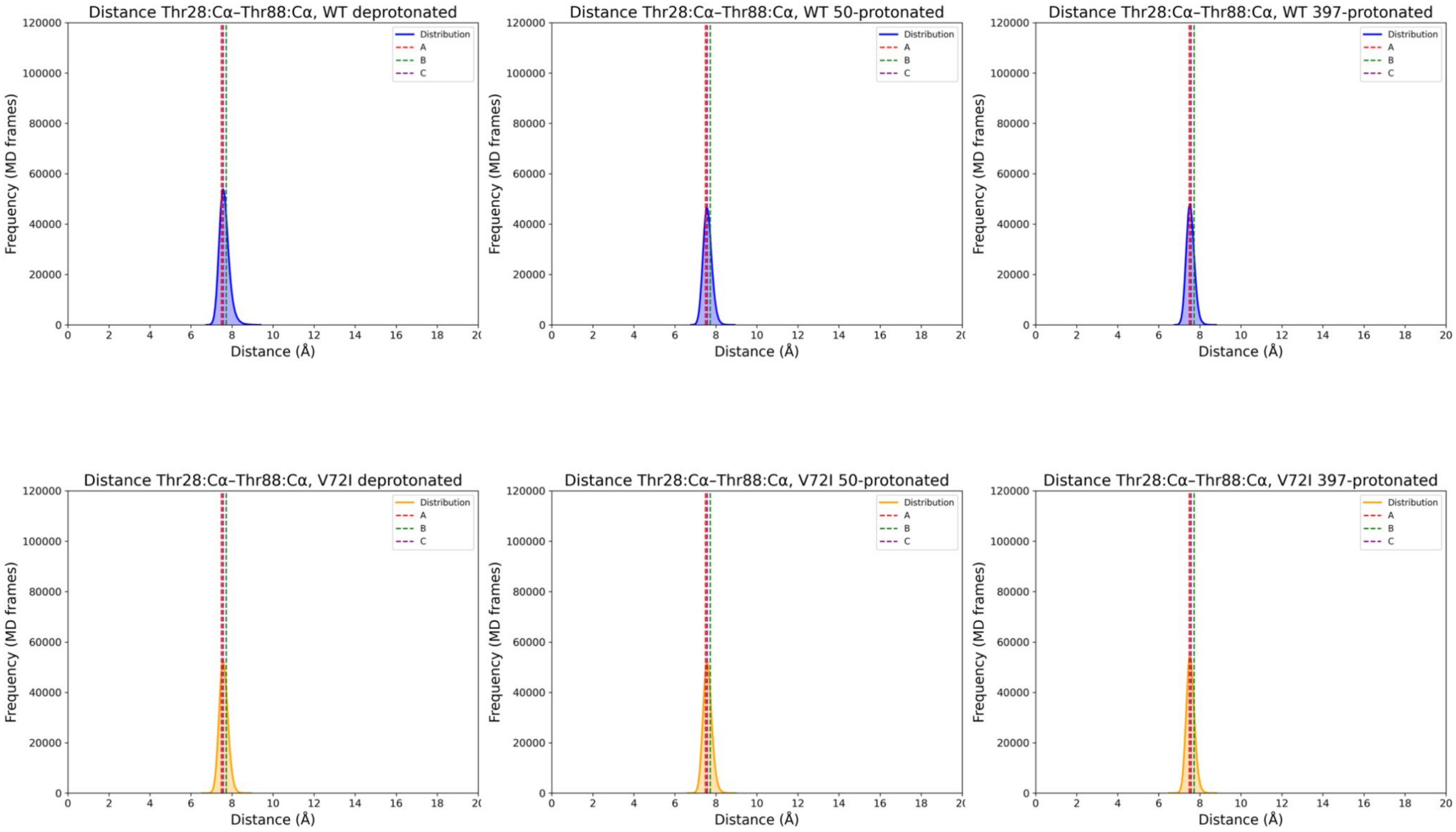

Figure S9. Histograms showing the distribution of the Thr28:Cα–Thr88:Cα distance during the course of our previously published MD simulations (L. Torielli *et al.*, *Nat. Commun.* **2025**, *16*, 3158) of: WT **D** (top; blue histograms); and V72I **D** (bottom; orange histograms); with: Asp50 and Asp397 both deprotonated (leftmost panels); Asp50 protonated (central panels); or Asp397 protonated (rightmost panels). Overlaid dotted lines denote the value of the same Cα–Cα distance in the MD frame from which we excided cluster models $\mathbf{A}_{\mathbf{prot}}$, $\mathbf{B}_{\mathbf{prot}}$, $\mathbf{C}_{\mathbf{prot}}$.

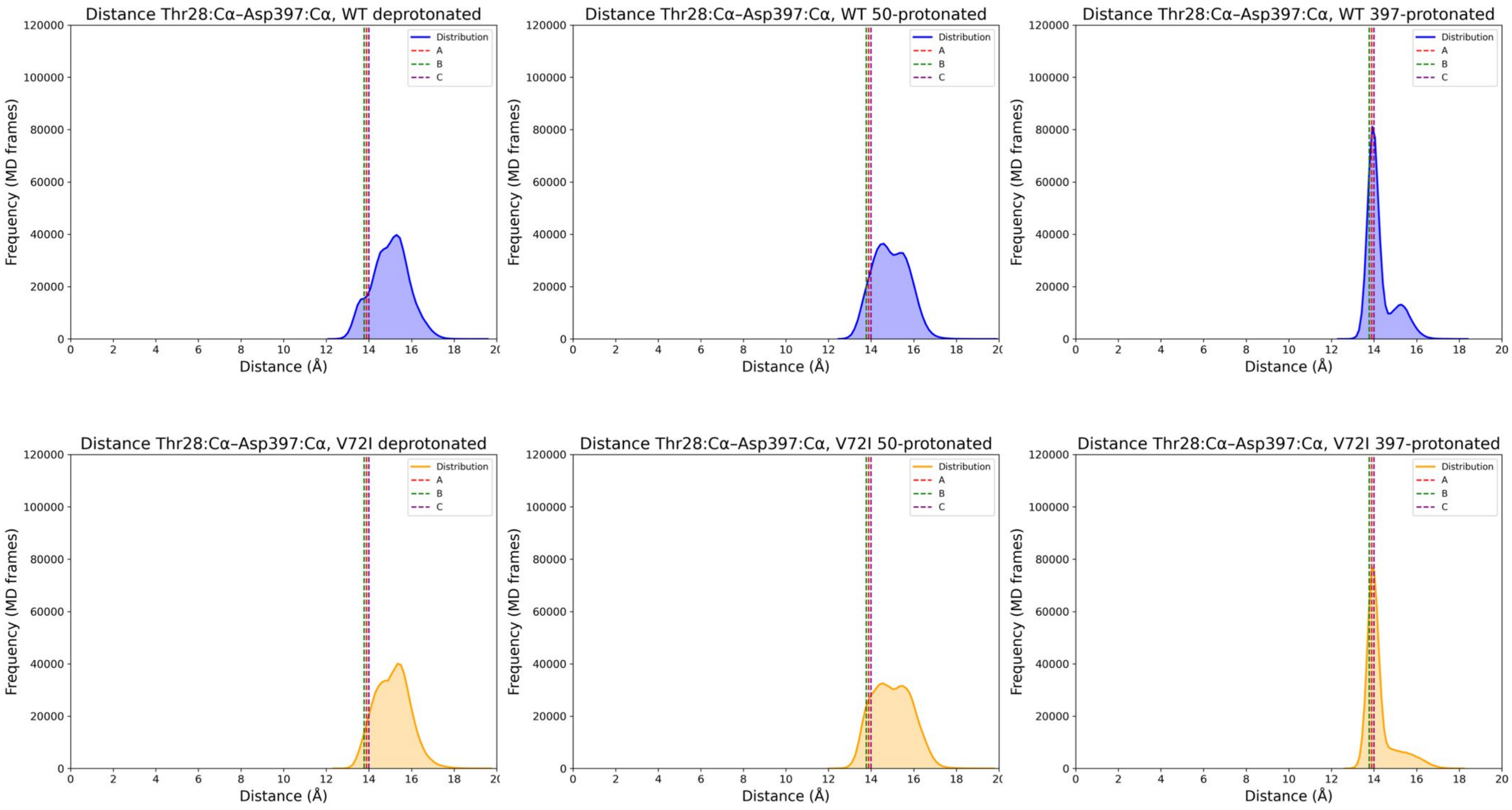

Figure S10. Histograms showing the distribution of the Thr28:Cα–Asp397:Cα distance during the course of our previously published MD simulations (L. Torielli *et al.*, *Nat. Commun.* **2025**, *16*, 3158) of: WT **D** (top; blue histograms); and V72I **D** (bottom; orange histograms); with: Asp50 and Asp397 both deprotonated (leftmost panels); Asp50 protonated (central panels); or Asp397 protonated (rightmost panels). Overlaid dotted lines denote the value of the same Cα–Cα distance in the MD frame from which we excided cluster models $\mathbf{A}_{\mathbf{prot}}$, $\mathbf{B}_{\mathbf{prot}}$, $\mathbf{C}_{\mathbf{prot}}$.

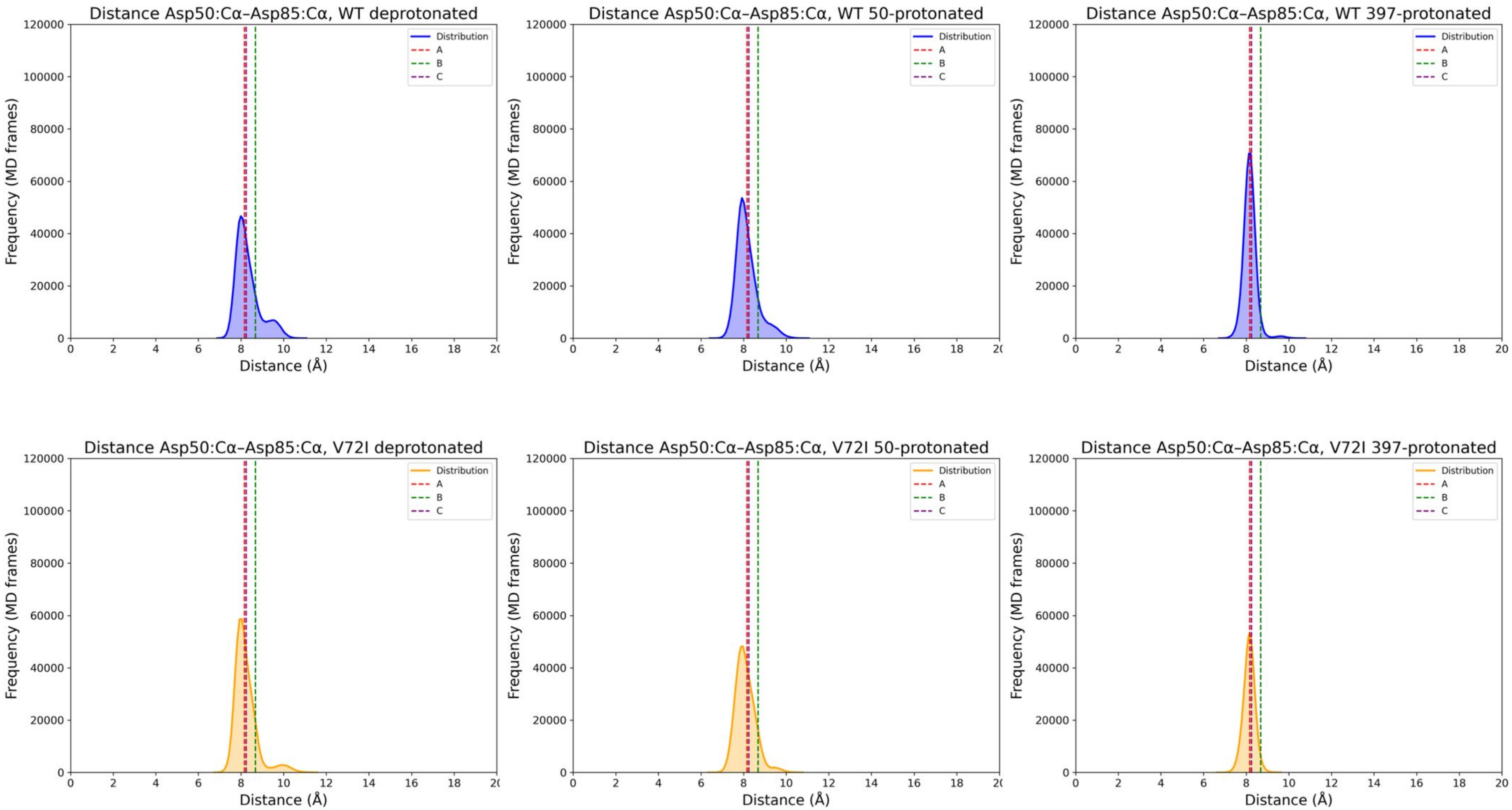

Figure S11. Histograms showing the distribution of the Asp50:Cα–Asp85:Cα distance during the course of our previously published MD simulations (L. Torielli *et al.*, *Nat. Commun.* **2025**, *16*, 3158) of: WT **D** (top; blue histograms); and V72I **D** (bottom; orange histograms); with: Asp50 and Asp397 both deprotonated (leftmost panels); Asp50 protonated (central panels); or Asp397 protonated (rightmost panels). Overlaid dotted lines denote the value of the same Cα–Cα distance in the MD frame from which we excided cluster models $\mathbf{A_{prot}}$, $\mathbf{B_{prot}}$, $\mathbf{C_{prot}}$.

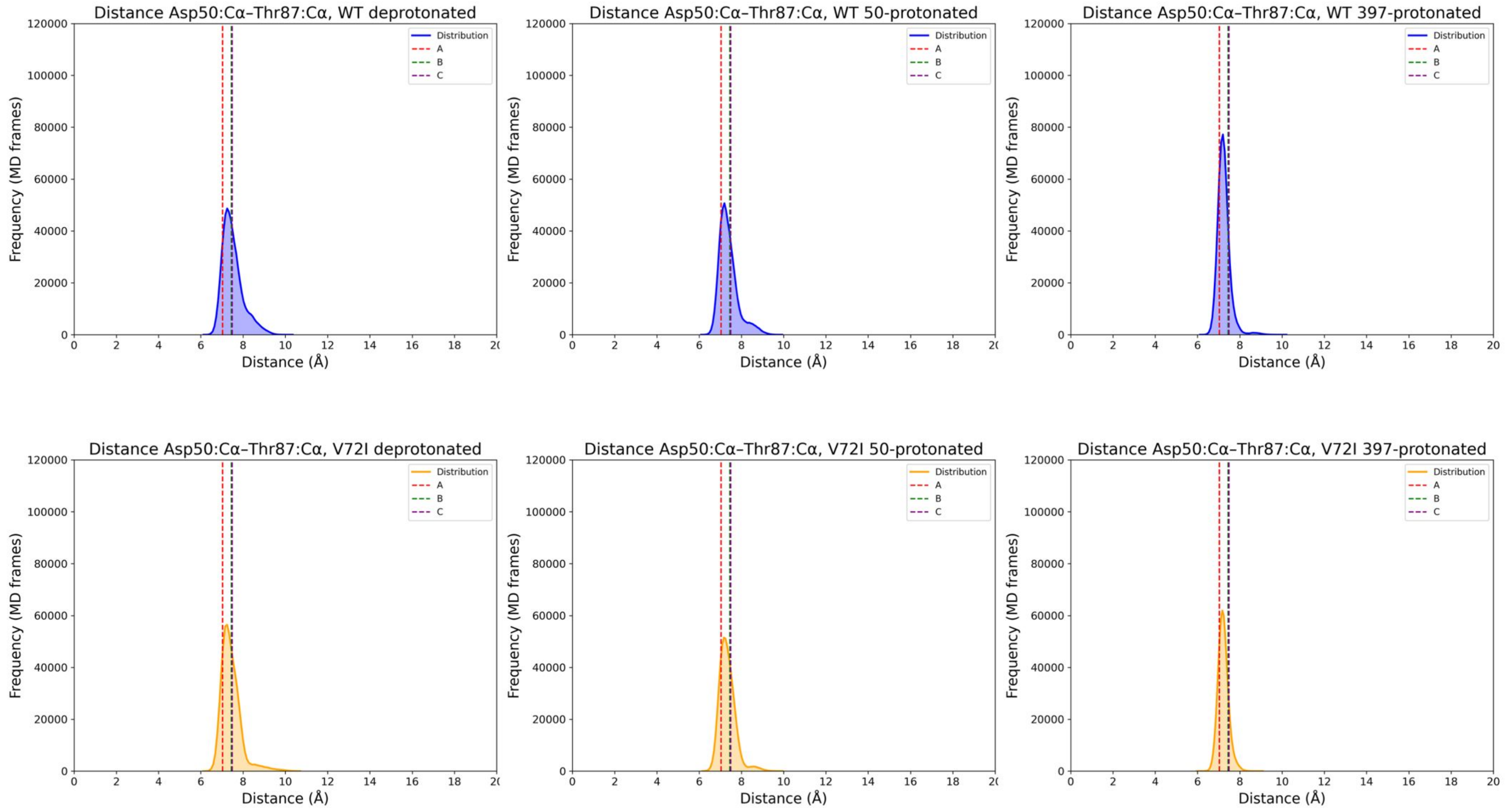

Figure S12. Histograms showing the distribution of the Asp50:Cα–Thr87:Cα distance during the course of our previously published MD simulations (L. Torielli *et al.*, *Nat. Commun.* **2025**, *16*, 3158) of: WT **D** (top; blue histograms); and V72I **D** (bottom; orange histograms); with: Asp50 and Asp397 both deprotonated (leftmost panels); Asp50 protonated (central panels); or Asp397 protonated (rightmost panels). Overlaid dotted lines denote the value of the same Cα–Cα distance in the MD frame from which we excided cluster models $\mathbf{A}_{\mathbf{prot}}$, $\mathbf{B}_{\mathbf{prot}}$, $\mathbf{C}_{\mathbf{prot}}$.

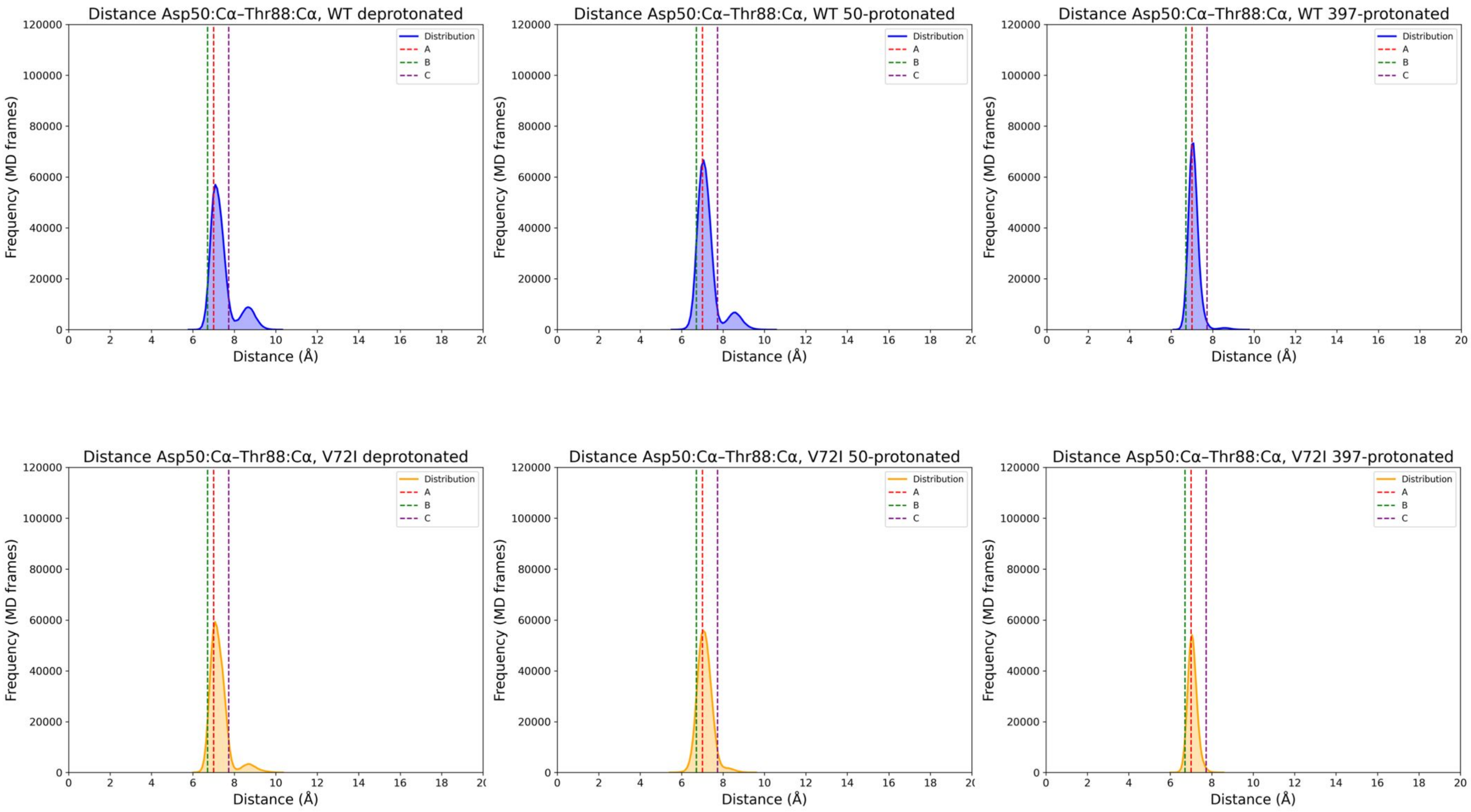

Figure S13. Histograms showing the distribution of the Asp50:Cα–Thr88:Cα distance during the course of our previously published MD simulations (L. Torielli *et al.*, *Nat. Commun.* **2025**, *16*, 3158) of: WT **D** (top; blue histograms); and V72I **D** (bottom; orange histograms); with: Asp50 and Asp397 both deprotonated (leftmost panels); Asp50 protonated (central panels); or Asp397 protonated (rightmost panels). Overlaid dotted lines denote the value of the same Cα–Cα distance in the MD frame from which we excided cluster models $\mathbf{A}_{prot}$, $\mathbf{B}_{prot}$, $\mathbf{C}_{prot}$.

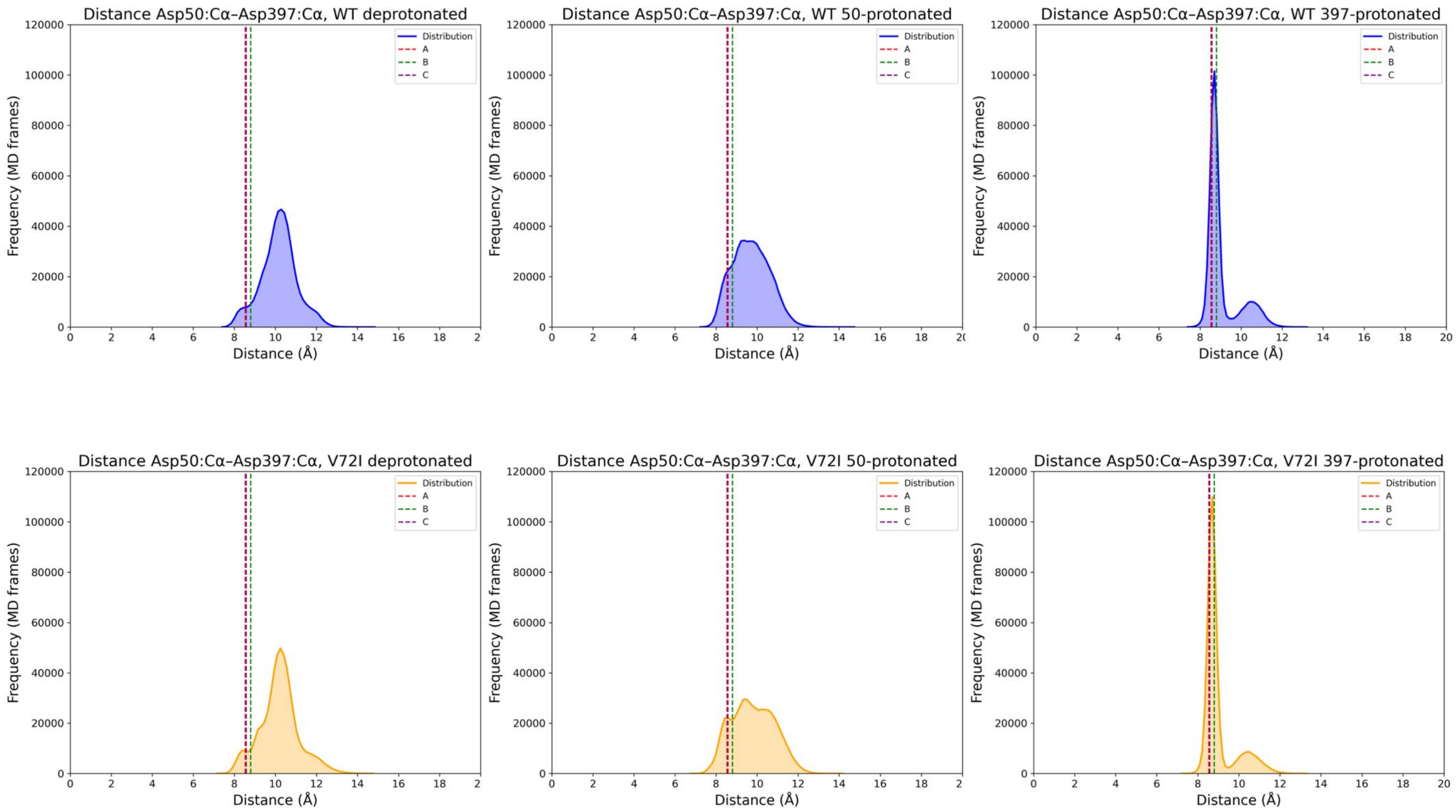

Figure S14. Histograms showing the distribution of the Asp50:Cα–Asp397:Cα distance during the course of our previously published MD simulations (L. Torielli *et al.*, *Nat. Commun.* **2025**, *16*, 3158) of: WT **D** (top; blue histograms); and V72I **D** (bottom; orange histograms); with: Asp50 and Asp397 both deprotonated (leftmost panels); Asp50 protonated (central panels); or Asp397 protonated (rightmost panels). Overlaid dotted lines denote the value of the same Cα–Cα distance in the MD frame from which we excided cluster models $\mathbf{A_{prot}}$, $\mathbf{B_{prot}}$, $\mathbf{C_{prot}}$.

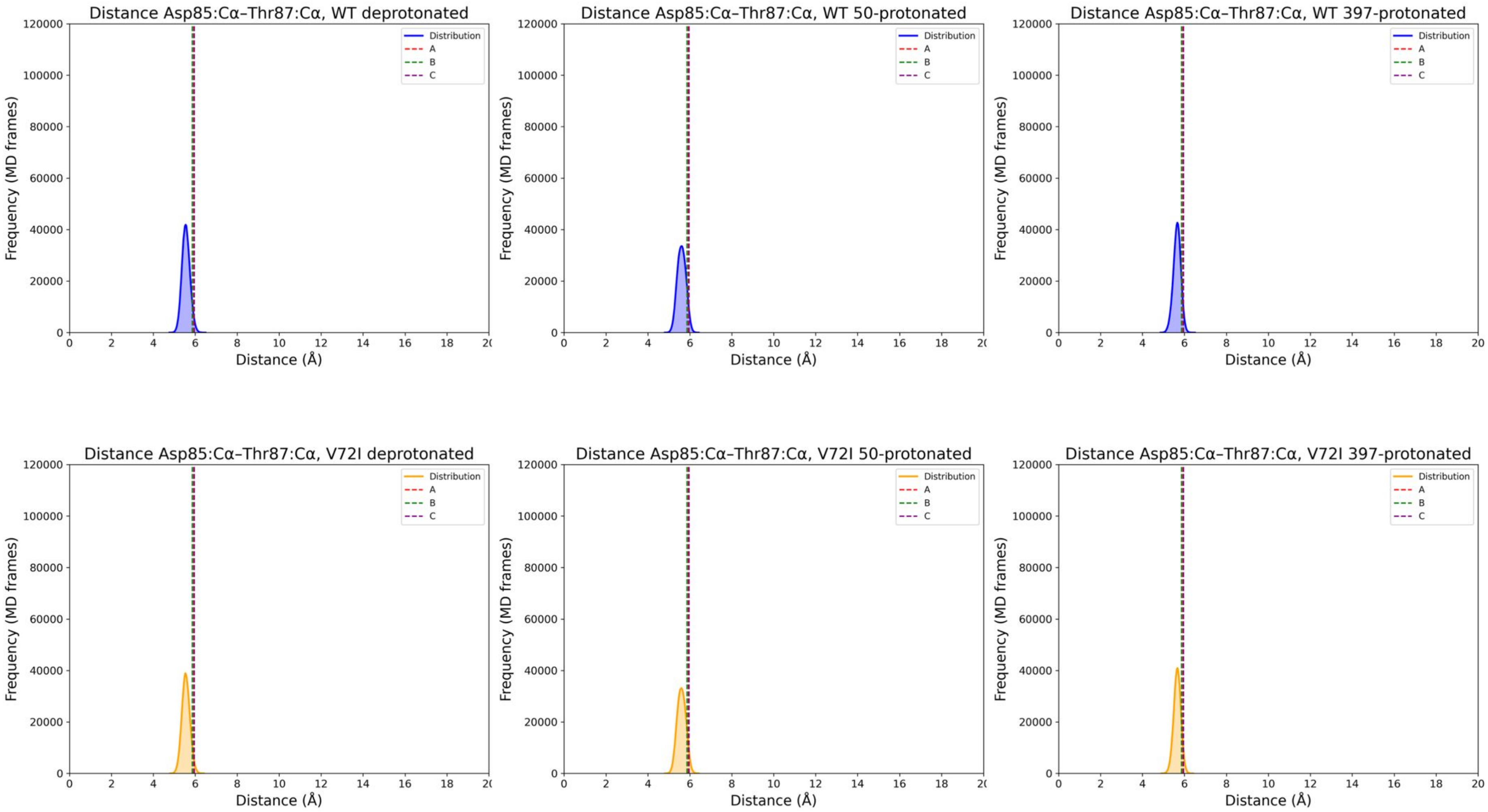

Figure S15. Histograms showing the distribution of the Asp85:Cα–Thr87:Cα distance during the course of our previously published MD simulations (L. Torielli *et al.*, *Nat. Commun.* **2025**, *16*, 3158) of: WT **D** (top; blue histograms); and V72I **D** (bottom; orange histograms); with: Asp50 and Asp397 both deprotonated (leftmost panels); Asp50 protonated (central panels); or Asp397 protonated (rightmost panels). Overlaid dotted lines denote the value of the same Cα–Cα distance in the MD frame from which we excided cluster models $\mathbf{A_{prot}}$, $\mathbf{B_{prot}}$, $\mathbf{C_{prot}}$.

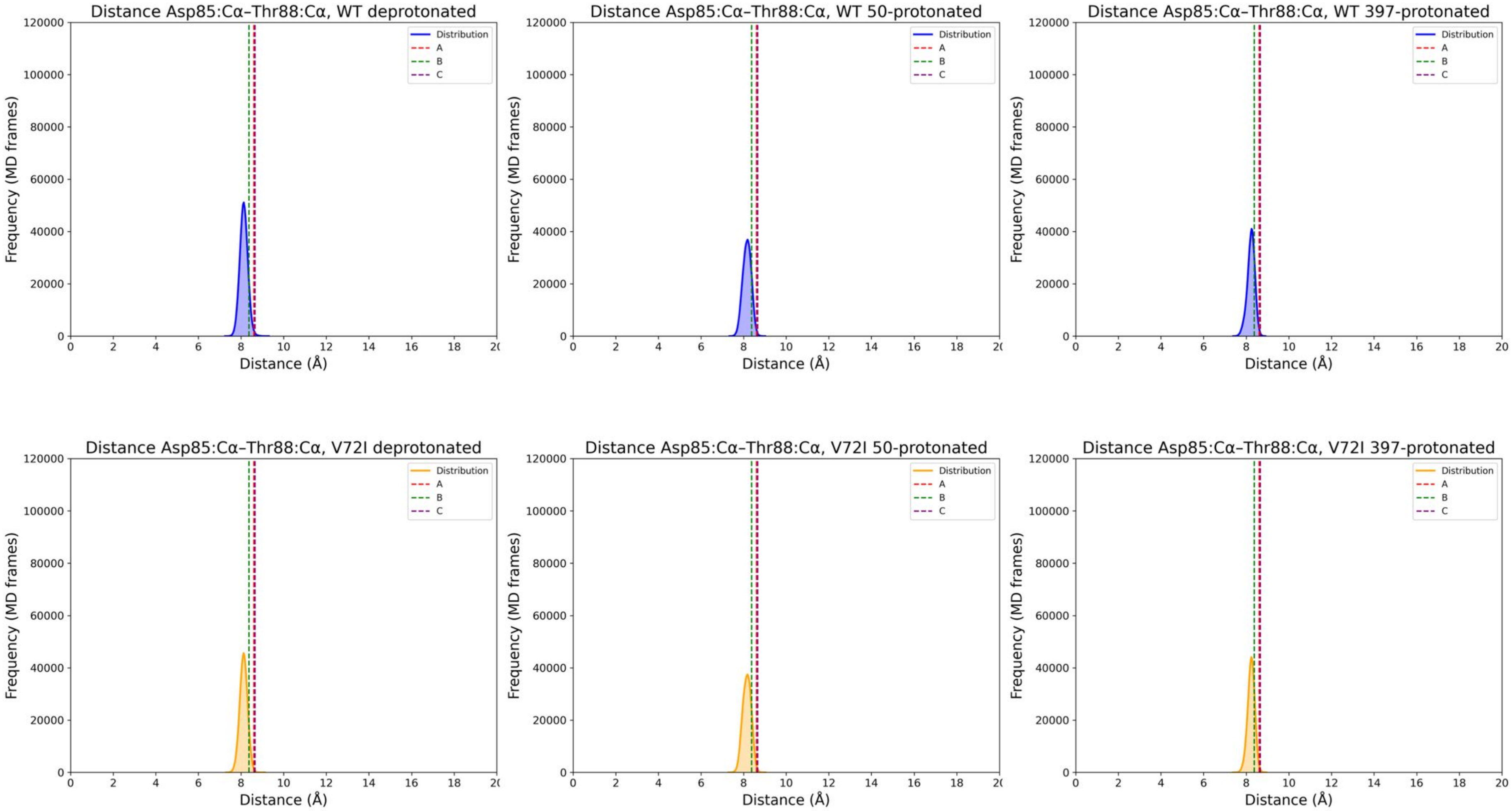

Figure S16. Histograms showing the distribution of the Asp85:Cα–Thr88:Cα distance during the course of our previously published MD simulations (L. Torielli *et al.*, *Nat. Commun.* **2025**, *16*, 3158) of: WT **D** (top; blue histograms); and V72I **D** (bottom; orange histograms); with: Asp50 and Asp397 both deprotonated (leftmost panels); Asp50 protonated (central panels); or Asp397 protonated (rightmost panels). Overlaid dotted lines denote the value of the same Cα–Cα distance in the MD frame from which we excided cluster models **A**$_{\mathbf{prot}}$, **B**$_{\mathbf{prot}}$, **C**$_{\mathbf{prot}}$.

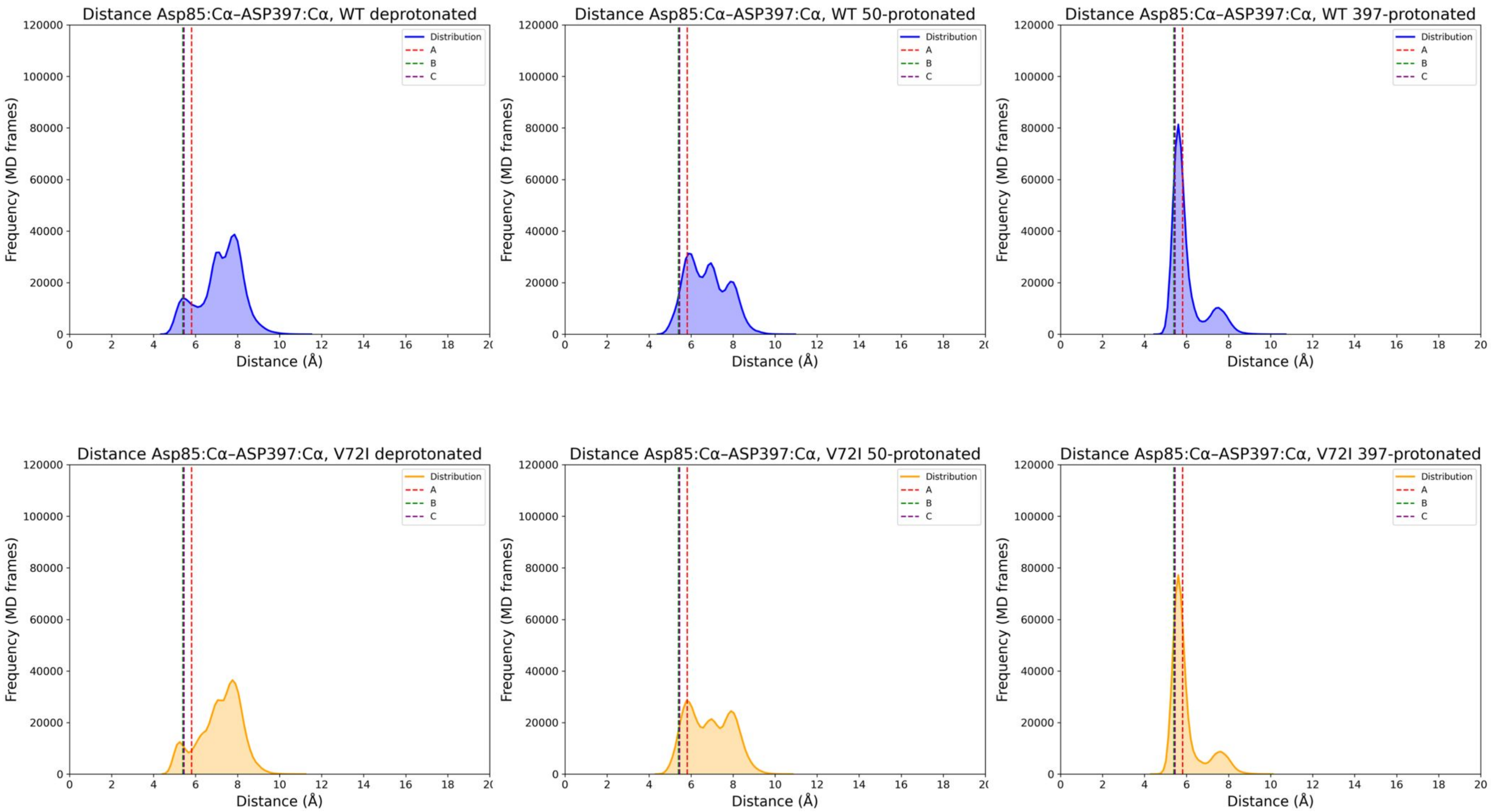

Figure S17. Histograms showing the distribution of the Asp85:Cα–Asp397:Cα distance during the course of our previously published MD simulations (L. Torielli *et al.*, *Nat. Commun.* **2025**, *16*, 3158) of: WT **D** (top; blue histograms); and V72I **D** (bottom; orange histograms); with: Asp50 and Asp397 both deprotonated (leftmost panels); Asp50 protonated (central panels); or Asp397 protonated (rightmost panels). Overlaid dotted lines denote the value of the same Cα–Cα distance in the MD frame from which we excided cluster models $\mathbf{A_{prot}}$, $\mathbf{B_{prot}}$, $\mathbf{C_{prot}}$.

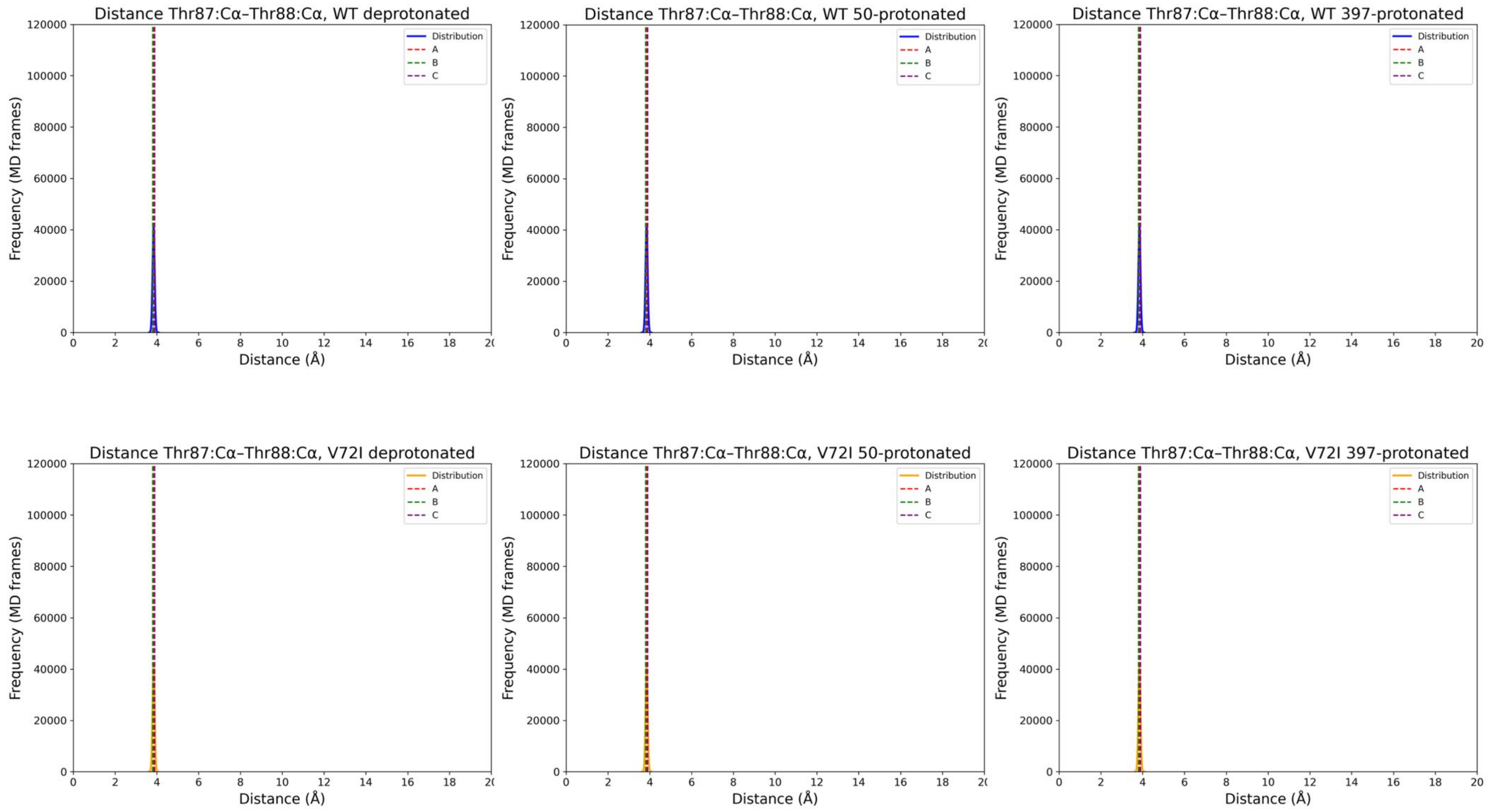

Figure S18. Histograms showing the distribution of the Thr87:Cα–Thr88:Cα distance during the course of our previously published MD simulations (L. Torielli *et al.*, *Nat. Commun.* **2025**, *16*, 3158) of: WT **D** (top; blue histograms); and V72I **D** (bottom; orange histograms); with: Asp50 and Asp397 both deprotonated (leftmost panels); Asp50 protonated (central panels); or Asp397 protonated (rightmost panels). Overlaid dotted lines denote the value of the same Cα–Cα distance in the MD frame from which we excided cluster models $\mathbf{A_{prot}}$, $\mathbf{B_{prot}}$, $\mathbf{C_{prot}}$.

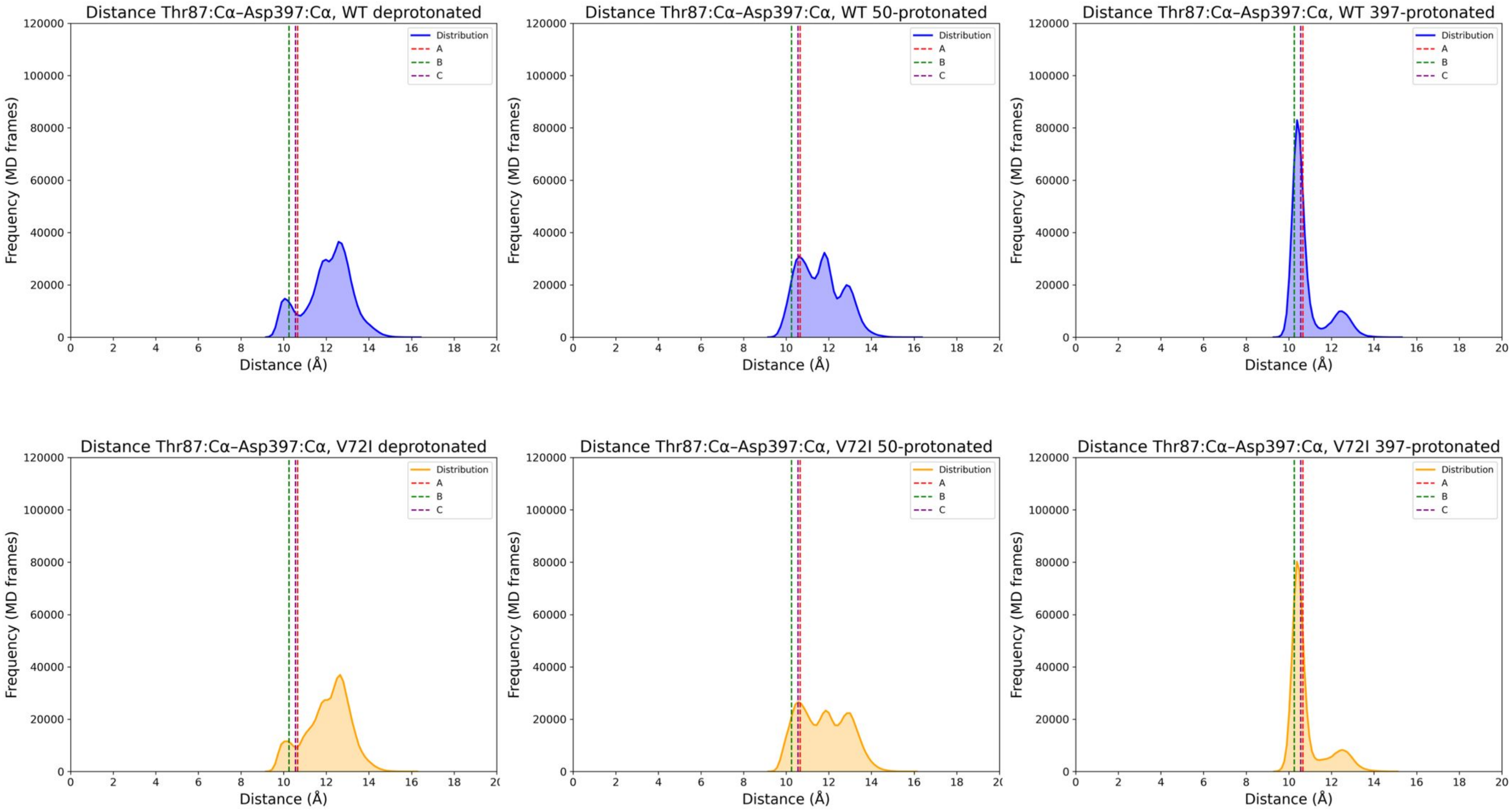

Figure S19. Histograms showing the distribution of the Thr87:Cα–Asp397:Cα distance during the course of our previously published MD simulations (L. Torielli *et al.*, *Nat. Commun.* **2025**, *16*, 3158) of: WT **D** (top; blue histograms); and V72I **D** (bottom; orange histograms); with: Asp50 and Asp397 both deprotonated (leftmost panels); Asp50 protonated (central panels); or Asp397 protonated (rightmost panels). Overlaid dotted lines denote the value of the same Cα–Cα distance in the MD frame from which we excided cluster models $\mathbf{A_{prot}}$, $\mathbf{B_{prot}}$, $\mathbf{C_{prot}}$.

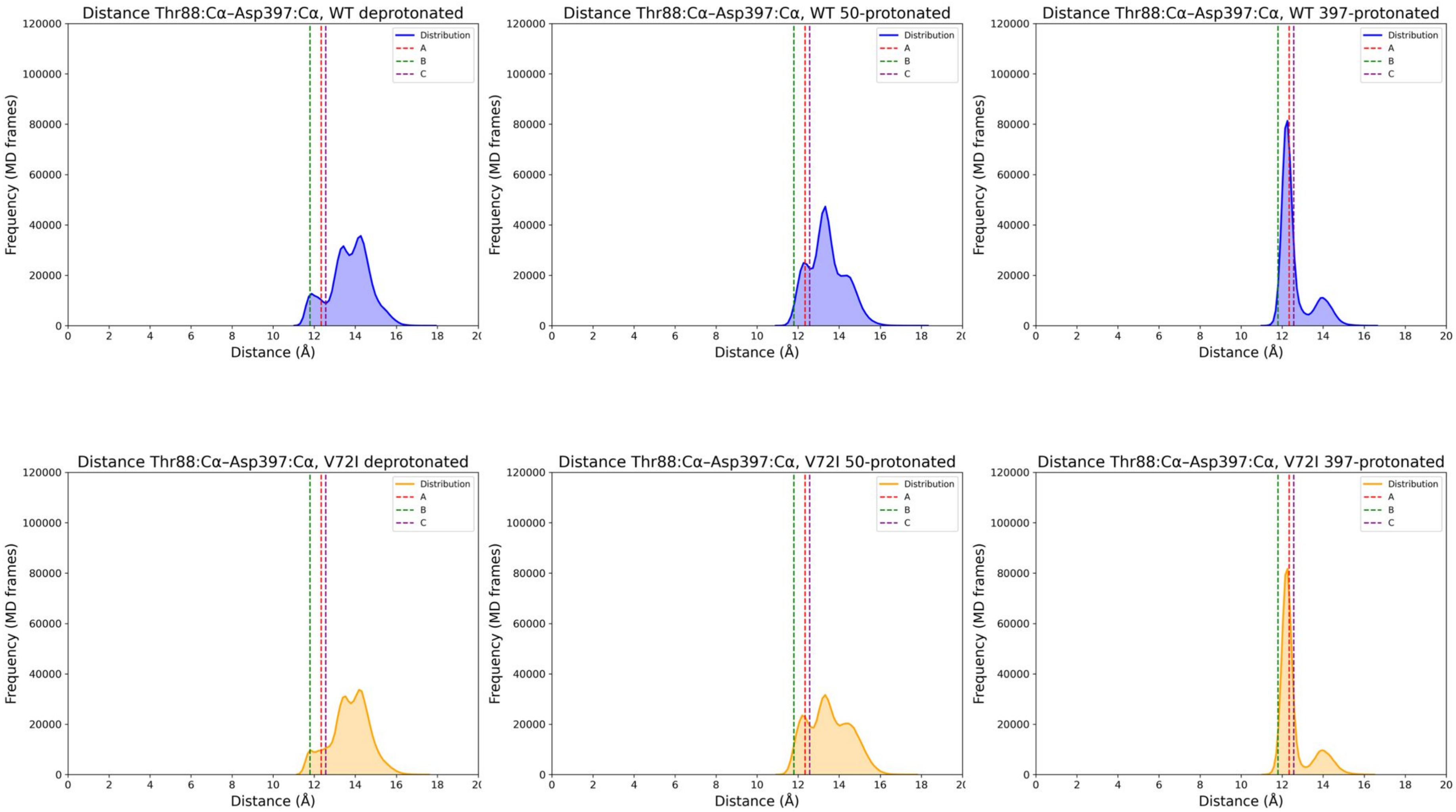

Figure S20. Histograms showing the distribution of the Thr88:Cα–Asp397:Cα distance during the course of our previously published MD simulations (L. Torielli *et al.*, *Nat. Commun.* **2025**, *16*, 3158) of: WT **D** (top; blue histograms); and V72I **D** (bottom; orange histograms); with: Asp50 and Asp397 both deprotonated (leftmost panels); Asp50 protonated (central panels); or Asp397 protonated (rightmost panels). Overlaid dotted lines denote the value of the same Cα–Cα distance in the MD frame from which we excided cluster models $\mathbf{A}_{\mathbf{prot}}$, $\mathbf{B}_{\mathbf{prot}}$, $\mathbf{C}_{\mathbf{prot}}$.
